# *Ecballium elaterium* (L.) A. Rich. (Squirting Cucumber) Plants Cultured Under Different Temperatures: Anatomical and Biochemical Modifications of Their Leaves and the Bioactivity of Leaf Extracts

**DOI:** 10.3390/metabo15090585

**Published:** 2025-08-31

**Authors:** Aikaterina L. Stefi, Maria Chalkiadaki, Emily Bashari, Konstantina Mitsigiorgi, Paweł Szczeblewski, Danae Papageorgiou, Dimitrios Gkikas, Dido Vassilacopoulou, Nikolaos S. Christodoulakis, Maria Halabalaki

**Affiliations:** 1 Section of Botany, Department of Biology, Faculty of Sciences, National and Kapodistrian University of Athens, 15784 Athens, Greece; kstefi@biol.uoa.gr (A.L.S.); milibashari@gmail.com (E.B.); mitsig@biol.uoa.gr (K.M.); danaepapageorgiou@gmail.com (D.P.); dimgkikas@biol.uoa.gr (D.G.); nchristo@biol.uoa.gr (N.S.C.); 2 Division of Pharmacognosy and Natural Products Chemistry, Department of Pharmacy, National and Kapodistrian University of Athens, 15784 Athens, Greece; mariachalk@pharm.uoa.gr; 3 Department of Pharmaceutical Technology and Biochemistry and BioTechMed Centre, Chemical Faculty, Gdansk University of Technology, 80-233 Gdansk, Poland; pawel.szczeblewski@pg.edu.pl; 4 Section of Biochemistry and Molecular Biology, Department of Biology, Faculty of Sciences, National and Kapodistrian University of Athens, 15784 Athens, Greece; didovass@biol.uoa.gr

**Keywords:** *Ecballium elaterium*, heat stress, LC-HRMS/MS, cytotoxic, SH-SY5Y cells, MCF-7 cells, DU-145

## Abstract

Background/Objectives: *Ecballium elaterium* is a widely distributed species and is one of the earliest recorded in traditional medicine. With global temperatures rising, this study aimed to investigate the changes in *E. elaterium* plantlets subjected to thermal stress. The goal was to understand how thermal stress affects morphology, physiology, and bioactive metabolite production, both for ecological adaptation and potential therapeutic applications. Methods: Seedlings were cultivated under controlled conditions and subjected to either the control temperature (22 °C) or the heat stress temperature (35 °C) for one week. Morphological and anatomical traits were assessed, along with physiological parameters such as chlorophyll content, malondialdehyde (MDA), hydrogen peroxide (H_2_O_2_), L-proline, soluble sugars, and total phenolic content. Methanolic leaf extracts from both groups were analyzed via LC-HRMS/MS and examined *in vitro* for cytotoxic activity against three human cancer cell lines: MCF-7 (breast), DU-145 (prostate), and SH-SY5Y (neuroblastoma). Results: Heat stress reduced dry mass and stomatal density but increased the diameter of the root transition zone, indicating anatomical adaptation. Leaves exhibited elevated oxidative stress markers and altered metabolite accumulation, while the roots showed a more integrated stress response. LC-HRMS/MS profiling revealed significant shifts in Cucurbitacin composition. Extracts from heat-stressed plants displayed stronger cytotoxicity, particularly toward DU-145 and SH-SY5Y cells, correlating with higher levels of glycosylated Cucurbitacins. Conclusions: *E. elaterium* demonstrates organ-specific thermotolerance mechanisms, with heat stress enhancing the production of bioactive metabolites. These stress-induced phytochemicals, especially Cucurbitacins, hold promise for future cancer research and therapeutic applications.

## 1. Introduction

*Ecballium elaterium* (L.) A. Rich., commonly known as squirting cucumber (Cucurbitaceae), is a monotypic plant species that naturally grows in the Mediterranean region. It is found on disturbed, stony ground in rubble and slopes near the sea and in disturbed habitats, such as roadsides, canal embankments, garbage dumps, and abandoned lots, as well as close to cultivations [1,2]. It is cultivated in Central Europe and England. It is classified as a geophyte, more specifically as a cryptophyte. It is an annual herb that is fleshy and rough/hairy, with stems 30–100 cm long, no tendrils, small yellow monoecious [3] or dioecious flowers [4], with a medium-sized oblong, watery, hairy berry ejecting seeds by elastic contraction [5]. The plant thrives in areas where Mediterranean climate imposes serious challenges to plants, forcing them to adapt in various ways to the unfavorable conditions of this environment. During their long-lasting convergent evolution, the plants have developed interesting adaptations and unique features [6,7,8,9,10,11,12,13,14].

The most peculiar and unusual characteristic of the squirting cucumber is its autochory seed dispersal, which also shapes its Latin binomial, *Ecballium elaterium* (*Ecballium* derives from the Greek verb *ἐκβάλλω*, meaning “to expel”). It refers to a mechanism that occurs due to the cells of the pericarp, which are under compression from high osmotic pressure. When they mature, they expand very rapidly, and the fruit loses its connection to the pedicel and detaches itself explosively, scattering seeds and juice out from the resulting basal opening [1,10].

In the era of Ancient Greece, the plant was referred to as “elaterium”, a source of important medical products, by Dioscorides [15], Theophrastus [16], Galenus Med., and Pliny [17]. The roots and fruit of this plant have been used in traditional medicine since ancient times in the Mediterranean basin. It is often used in purgatives; as a fresh juice applied locally to treat hemorrhoids, varicose veins, and nosebleeds; in dropsy (edema), especially pulmonary edema; and as a revulsive in brain diseases [2]. Other traditional uses include the treatment of constipation, agglutination of erythrocytes, hypertension, inflammation, modification of the heart rate [18], cancer, fever, liver cirrhosis, rheumatic disease, and malaria in humans [2]. Many of the biological activities of this species, such as its antioxidant, antibacterial (against both Gram-positive and Gram-negative bacteria), anti-inflammatory, cytotoxic, antimalarial, antiviral, and wound-healing activities, have been attributed to a type of triterpene called Cucurbitacins, especially A, E, and I, and their glycosylated derivatives [19,20,21]. It is important to note that irresponsible use of the plant may cause toxic, allergic, and systemic adverse reactions. The fruit of this plant is poisonous and exhibits its toxic activity on the respiratory, cardiac, and gastric systems and on mucous membranes [5].

The Mediterranean basin is recognized as one of the most climate-change-vulnerable regions globally, experiencing rising temperatures, prolonged heatwaves, and increased frequency of extreme weather events [22,23]. These climatic shifts pose significant threats to the native flora, particularly to species growing in semi-arid and arid environments where temperature stress directly impacts plant physiology, productivity, and survival. *Ecballium elaterium*, which has thrived in and naturally adapted to this environment for more than two millennia, offering its medicinal properties to humans across the Mediterranean, serves as an ideal model for understanding its potential thermotolerance within the frame of climate crisis.

This study builds upon our previous work, which established the baseline morphological traits of *Ecballium elaterium* leaves under normal environmental conditions [10]. By using that morphological baseline, we now investigate how a 10-day heat stress event—designed to simulate the short-term heatwaves that are increasingly common in the Mediterranean basin due to climate change—affects both the morphological and biochemical characteristics of the leaves. This approach allows us to assess the acute physiological responses of the plant, such as changes in antioxidant enzyme activity, secondary metabolite biosynthesis, and their bioactivity. Understanding these responses is crucial for predicting how *E. elaterium* may adapt and/or respond to future climatic challenges. Moreover, this research provides valuable insight into how elevated temperatures might influence the plant’s bioactive compounds and thus its therapeutic potential under evolving environmental conditions.

## 2. Materials and Methods

### 2.1. Plant Material and Experimental Setup

**Figure 1:**
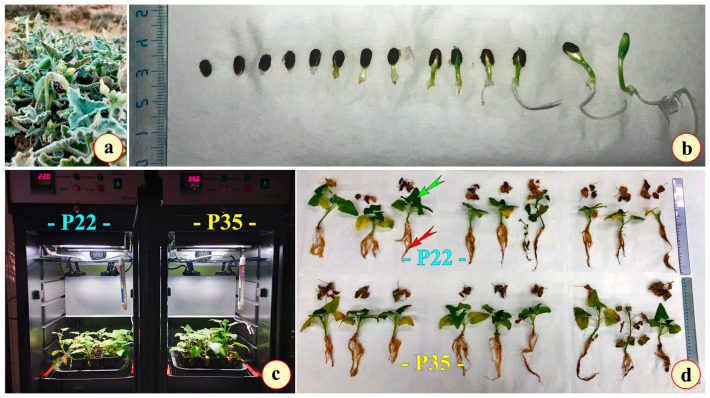
The experimental setup. (**a**) The wild growing plant in its natural habitat. A rare snapshot by the time of non-proper rupture of the fruit. (**b**) Germinated seeds at various stages of their development. (**c**) The two culture chambers; the temperature is indicated in the upper part of each panel; it was adjusted to 22.0 °C for P22 (left chamber) and to 35.0 °C for P35 (right chamber). (**d**) The two groups of cultured plants, after the end of the first experiment; the upper line is for P22 while the lower line is for P35; arrows indicate the part of the plant that was sampled.

Seeds of *E. elaterium* were collected from a hill slope on the Aegean Island of Ios (Greece) (36.74618 N, 25.27343″ E; altitude 220 m) in mid-August 2023 (Figure 1a). They were placed in a P-Selecta incubator (Model No. 2000238, Barcelona, Spain) with low temperature (17 °C) and humidity (30%), along with silica gels, and were allowed to dry until late September 2023. Then, the seeds were imbibed in Eppendorf tubes with distilled water at room temperature for 24 h in order to initiate germination. Afterwards, they were placed on a Petri dish with two sheets of filter paper with the addition of 3 mL of distilled water, and incubated at 20 °C, with 70% humidity, in a light/dark cycle of 16 h/8 h. The germination process was completed in 12 days, achieving a germination rate of 78%. When the radicle of the germinated seeds protruded at about 1 cm (Figure 1b), the seedlings were transferred to small culture pots (70 × 70 mm pots; 400 mL volume) containing Potgrond P medium (frozen through black peat (Klasmann-Deilmann, Geeste, 49744, Germany) and organic matter 90–95% (*w*/*w*). The conditions were as follows: humidity, 60–70% (*w*/*w*); conductivity, 40 mS/m (±25%); pH (H_2_O), 5.5–6.5; NPK Fertilizer (14:10:18), 1.5 kg/m^3^. A total of 18 pots were used (9 for each treatment; 1 young plant was accommodated in each pot). Afterwards, they were placed in a culture chamber (Elvem—model BOD100; BOD100—Spata 19004; Athens, Greece) under controlled conditions (temperature 22 °C, humidity 70%, photoperiod in a light/dark cycle of 16 h/8 h). The chambers are equipped with built-in illumination sources: two sources of 1000 lumen, with 10W LED at 4500 K, assisted by two Osram basic (Osram GmbH, Munich, 80807, Germany), T5 Short, and L 6W/640 miniature tubes (length, 212 mm; diameter, 16 mm; 4000 K in each chamber). The total luminance at the surface of the pots, directly measured with a MASTECH MS6610 portable luxmeter, was 125.7 to 128 μmol m^−2^ s^−1^. The chambers were ventilated through a HAILEA ACO-9160 (Guangdong Hailea Group Co., Ltd., Chaozhou Guangdong, 515700, China), at an output of 4 L/min. The plants remained there for approximately 8 weeks (October 2023–December 2023).

In December 2023, two months after initial planting, the young plants were divided into two groups, each comprising 9 pots, and transferred to trays placed in a temperature-controlled culture chamber to begin the thermal experiment (Figure 1c). One group was maintained at 22 °C (plants at 22 °C—P22), while the other group was subjected to a higher temperature of 35 °C (plants at 35 °C—P35). Both groups were kept under identical conditions of 70% relative humidity and a 16 h light/8 h dark photoperiod. To guarantee uniform exposure to light within the culture chamber, the positions of the trays were rotated daily. At 9:00 each morning, tray locations were shifted so that each spent time at the left, center, and right positions within the chamber over the course of the experiment. Applying this temperature over a span of 10 days simulates a prolonged heat event, which aligns with naturally occurring thermal extremes in the region and allows for the assessment of plant responses under stress conditions reflective of actual environmental scenarios. The selection of 35 °C for 10 days as a thermal stress condition for the plant is justified based both on the ombrothermic diagrams, in particular that of the year 2022, and on the temperature data for July and August from 2018 to 2023 (the sampling year) (see Supplementary Figures). Relative humidity (RH) was continuously monitored with a Xiaomi Monitor 2–Mi (NUN4126GL—Xiaomi Corporation, Haidian District, Beijing 100085, China) in each chamber and recorded at regular intervals. RH remained stable within chambers during the experiment (P35 chamber ~65%; P22 chamber (30%). After the 10-day thermal stress, the pots were removed from the chamber. The culture medium was gently immersed in water to free the seedlings, and their roots were carefully washed to eliminate all traces of the Potgrond P substrate (Figure 1d). Plant tissues—including leaves and roots—were then collected and weighed for use in fixation and photosynthetic pigment analysis, while samples selected for the hydrogen peroxide (H_2_O_2_) and malondialdehyde (MDA) measurements were rapidly frozen in liquid nitrogen and stored at −80 °C until analysis. Finally, the plant samples were placed on filter paper and dried in an oven at 60 °C for 72 h. These dried materials were subsequently used for the extraction and quantification of phenolic compounds, soluble sugars, and L-proline content.

The thermal experiment was conducted a second time to verify the accuracy of the initial findings. The experimental setup remained the same, with incubation duration of roughly eight weeks—from February to April 2024—preceding the application of thermal stress. On the first day that stress was introduced, all leaves selected from both treatments were tagged. These leaves began as young leaflets and had matured into fully developed foliage by the time they were collected.

### 2.2. Microscopy

At the end of the experiment, small square parts (1 × 1 mm) of the leaf tissue, excluding the central nerve, were removed at random from the middle of the upper pair of leaves (of each of the 9 plants; aged more than 10 days), and samples from primary roots (from 9 plants; 1 cm above the root tip) were cut. The leaf and root tissues were fixed in phosphate-buffered 3% glutaraldehyde (Merck KGaA, Darmstadt, Germany—pH 6.8) at 0 °C for 2 h and post-fixed in phosphate-buffered 1% osmium tetroxide (Merck KGaA, Darmstadt, Germany). They were then dehydrated in graded ethanol series. The prepared tissues were subjected to one of the following treatments: (a) Transferred in 100% acetone, critical point dried (Autosamdri^®^-815, Tousimis, Rockville, MD, USA), double-coated with gold and platinum, and viewed with a JEOL JSM-6360 high-vacuum scanning electron microscope (Tokyo, Japan); here, all electron micrographs were taken with the instrument’s built-in camera (accelerating voltage 20 kV; spot size 40). (b) Dehydrated in absolute ethanol, transferred in propylene oxide, and embedded in gradually increasing concentration of Durcupan ACM (Fluka Chemie GmbH, St. Gallen, Switzerland) (four component epoxy resin); finally, the tissue was left in pure Durcupan to polymerize at 68 °C for 48 h. Semi-thin sections were obtained with glass knives using an LKB Ultrotome III (LKB Bromma, SE-192 06 Sollentuna, Sweden), placed on glass slides, and stained with 0.5 toluidine blue O (in 1% borax solution), as a general stain, for light microscopic observations [24]. Sections of fresh or epoxy-embedded material were viewed with an OLYMPUS CX-41 Light Microscope (Olympus Corporation, Tokyo 192-8507, Japan). Original light micrographs were recorded digitally, using a Nikon D5600 camera (Nikon Corporation, Tokyo 140-8601, Japan) at 24.2 megapixels [25]. Secretive hair and stomatal frequencies were measured on the adaxial and abaxial epidermis of 5 mature leaves, in 30 different optical fields of the same magnitude for each leaf. For these measurements, scanning electron micrographs were used. The average stomatal frequencies and standard deviations were calculated. A one-way ANOVA was performed to assess the significant differences in stomatal frequencies among the groups. Following a significant ANOVA result, a Tukey’s post hoc HSD test was conducted to identify pairwise differences between groups. The fixation process was repeated in a second experiment to compare and validate the results, using the same methodology as previously described.

### 2.3. Histochemistry

Histochemical analysis was conducted on leaf and root sections using plastic-embedded tissue samples, with the objective of identifying specific secondary metabolites of interest. The following reagents were applied to semithin sections of the embedded material:

(a) A saturated solution of Sudan Black B in 70% ethanol, used to visualize lipid deposits [26].
(b) A saturated solution of Alcian Blue in 3% acetic acid, employed to detect stored polysaccharides [27].
(c) A 1% solution of Aniline Blue Black in 70% acetic acid, used for the histochemical identification of accumulated proteins [28].

All prepared slides were examined under an OLYMPUS CX41 light microscope.

### 2.4. Pigments Protocol

The total content of chlorophyll a (Chl-a), chlorophyll b (Chl-b), and carotenoids were determined using spectrophotometric analysis. Approximately 50 mg of fresh leaf tissue from each of 10 samples per treatment were extracted in 1 mL of 80% (*v*/*v*) acetone and stored at 4 °C for 48 h. Then, the extract was transferred into 1.5 mL glass cuvettes and analyzed using a V-1200 spectrophotometer (Avantor® delivered by VWR, Radnor, PA 19087, USA). Absorbance readings were taken at 470 nm for carotenoids, 663 nm for Chl-a, and 645 nm for Chl-b. Pigment concentrations were calculated using specific molar extinction coefficients appropriate for this method and normalized to fresh weight (mg/g), as described by [25]. The extinction coefficients used were as follows: for chlorophyll a, ε_663.6_ = 76.79 and ε_646.6_ = 18.58; for chlorophyll b, ε_663.6_ = 9.79 and ε_646.6_ = 47.04 [29,30,31]. All values were reported as the mean of two replicates per treatment (*N* = 10, *n* = 2) ± the standard error of the mean. Prior to statistical analysis, data were tested for normality, using the Shapiro–Wilk test. Differences among treatments were evaluated for statistical significance using Tukey’s test, performed with OriginPro v9.1 (OriginLab Corporation, Northampton, MA 01060, USA) and Microsoft Excel (Microsoft Corporation, Redmond, WA 98052-6399, USA). Principal Component Analysis (PCA) was subsequently applied to the dataset (see Section 2.14).

### 2.5. Protocol for MDA (Malondialdehyde) and H_2_O_2_ Determination in Plant Tissues

Reactive oxygen species (ROS) levels were indirectly assessed by measuring lipid peroxidation through the malondialdehyde (MDA) assay. For this purpose, 50 mg of plant material—previously frozen and ground in liquid nitrogen—was extracted with 1 mL of 0.25% thiobarbituric acid (TBA; Sigma-Aldrich™, St. Louis, MO 63103, USA) prepared in 10% trichloroacetic acid (TCA; Sigma-Aldrich™, St. Louis, MO 63103, USA). The extract was collected in a 1.5-mL eppendorf tube, and the mixture was heated at 85 °C for 30 min, and then quickly chilled on ice. The same procedure was followed for the blank sample but without plant material. The mixture was centrifuged at maximum speed (14,000 rpm—Eppendorf 5453 Minispin Plus Centrifuge, Eppendorf SE, 22339 Hamburg, Germany) for 10 min to pellet the particles. The supernatant was then transferred to a 1-mL plastic cuvette for spectrophotometric measurement. The absorbance was determined primarily at 532 nm (the peak of the MDA–TBA complex) and then at 600 nm (nonspecific absorption). A sample of 1 mL 0.25% TBA in 10% TCA was used as blank. A_(532–600)_ was calculated [32]. The MDA concentration was estimated using the Beer–Lambert–Bouguer law, MDA extinction coefficient ɛ_532_ = 155 mM^−1^ cm^−1^; the amount of MDA was calculated, and the values were normalized to the fresh weight of each sample.

Furthermore, oxidative stress was also measured by the H_2_O_2_ method [33,34]. Measures of 50 mg of leaf and root tissues were selected randomly as described above, and were harvested and homogenized in an ice bath with 0.5 mL 0.1% (*w*/*v*) trichloroacetic acid (TCA). The homogenate was centrifuged at 12,000× *g* for 15 min and then 0.5 mL of the supernatant was added to 0.5 mL 10 mM potassium phosphate buffer (pH 7.0); 1 mL 1 M potassium iodide (KI) was also added. The absorbance of supernatant was read at 390 nm, while the H_2_O_2_ content was calculated via a standard curve. For each treatment, two independent biological replicates were performed using separately cultivated plants. In each replicate, nine individual leaves/roots were collected and analyzed separately (*N* = 9). The data were first averaged within each replicate, and final results are expressed as the mean of two biological replicates (*n* = 2) ± standard error of the mean (SEM). Data were previously checked for their normality, while Tukey’s test was evaluated using OriginPro v.9.1 and MS Excel, for the statistical significance. Then, data was subjected to PCA analysis (see Section 2.14).

### 2.6. Determination of Total Phenolic Content

Total phenolic content (TPC) in leaf extracts was quantified using the Folin–Ciocalteu colorimetric assay [35,36]. For each treatment, 0.1 g of dry tissue was collected from 10 leaf samples, ground using a Mikro-Feinmühle-Cullati electric mill (IKA-Werke GmbH & Co. KG, 79219 Staufen, Germany), and suspended in 10 mL of 50% (*v*/*v*) methanol. The mixture was incubated in a water bath at 40 °C for 3 h, with periodic vortexing to facilitate extraction. Following incubation, the extracts were filtered through Whatman^®^ No. 2 filter paper, and the resulting solutions were stored in tightly sealed containers at 4 °C overnight for preservation of phenolic compounds. Furthermore, an aliquot (0.05 mL) of the diluted leaf extract (1:5 MeOH 10% (*v*/*v*)) was added to a solution consisted of 3.95 mL of dH_2_O, 0.25 mL Folin–Ciocalteu (Sigma-Aldrich™, St. Louis, MO 63103, USA) reagent (that was previously diluted with water 1:10 *v*/*v*), 0.75 mL Na_2_CO_3_ 20% (*w*/*v*) and was vortexed for 30 s. The solution was kept at 20°C for 2 h and the absorption of the resulting colorimetric reaction was measured with a Novaspec II UV–VIS spectrophotometer (Amersham Pharmacia Biotech AB, SE-751 84 Uppsala, Sweden) at 760 nm. Calculation of the total phenolic content was performed using standard curves of tannic acid and expressed as mg of tannic acid equivalent per g (dry weight) of leaf tissues. The standard curve is described in [37]. For each treatment, two independent biological replicates were performed using separately cultivated plants. In each replicate, nine individual leaves/roots were collected and analyzed separately (*N* = 9). The data were first averaged within each replicate, and final results are expressed as the mean of two biological replicates (*n* = 2) ± SEM. Data were previously checked for their normality, while Tukey’s test was conducted using OriginPro v.9.1 and MS Excel, to determine statistical significance. Then, the data were subjected to PCA analysis (see Section 2.14).

### 2.7. Determination of Total Soluble Sugar

Soluble sugars were extracted from dry, finely powdered samples leaves and roots that were placed in 10 mL 80% (*v*/*v*) ethanol, in a shaker, and the extracts were filtered using Whatman ^®^ (Merck KGaA, Darmstadt, 64293, Germany—Catalogue number-Cat. no. WHA1002150) filter paper. In three test tubes, 1 mL of the sample solution was added to each. Subsequently, 1 mL of phenol solution (Merck KGaA, Darmstadt, 64293, Germany-Cat. No. P9346) was added to each tube, and the tubes were incubated in a water bath at 20 °C for 20 min. After incubation, 5 mL of sulfuric acid (H_2_SO_4_) was added to each test tube, followed by vortex mixing. Finally, Soluble sugar concentration was measured at 490 nm using a V-1200 spectrophotometer (Avantor® delivered by WVR, Radnor, PA 19087, USA)) [38,39]. D-glucose (Sigma-Aldrich™, St. Louis, MO 63103, USA-Cat. no.50-99-7) was used to prepare aqueous solutions for the standard curve, while the results obtained from the analysis are expressed as mg g^−1^ dry weight. For each treatment, two independent biological replicates were performed using separately cultivated plants. In each replicate, nine individual leaves/roots were collected and analyzed separately (*N* = 9). The data were first averaged within each replicate, and final results are expressed as the mean of two biological replicates (*n* = 2) ± SEM. Data were previously checked for their normality, while Tukey’s test was conducted using OriginPro v.9.1 and MS Excel, for the statistical significance. Then, the data were subjected to PCA analysis (see Section 2.14).

### 2.8. Determination of Proline

Free proline content was determined colorimetrically using 4 mL samples of dried plant material [40,41,42]. Dried samples were ground into a fine powder under liquid nitrogen and homogenized in 20 mL of 3% (*v*/*v*) aqueous sulphosalicylic acid (Merck KGaA, Darmstadt, 69293, Germany-Cat. no. 8.18731). The homogenate was filtered through Whatman^®^ filter paper (Merck KGaA, Darmstadt, 64293, Germany-Cat. no. WHA1002150). A 2 mL aliquot of the filtrate was mixed with 2 mL of acid-ninhydrin solution (Sigma-Aldrich™, St. Louis, MO 63103, USA—151173) and 2 mL of glacial acetic acid in a test tube. The mixture was incubated at 100 °C in a water bath for 1 h. The reaction was then terminated by cooling in an ice bath. Following this, 4 mL of toluene (Sigma-Aldrich™, St. Louis, MO 63103, USA—650579) was added to extract the chromophore. The solution was vortexed and the upper toluene phase was carefully separated. The absorbance of this phase was measured at 520 nm using toluene as a blank on a V-1200 spectrophotometer (Avantor® delivered by WVR, Radnor, PA 19087, USA). Proline concentration was calculated based on a standard curve of L-proline (SERVA Electrophoresis GmbH, Heidelberg, 69115, Germany-Cat. no. 33582.02) and expressed as μmol g^−1^ dry weight. For each treatment, two independent biological replicates were performed using separately cultivated plants. In each replicate, nine individual leaves/roots were collected and analyzed separately (*N* = 9). The data were first averaged within each replicate, and final results are expressed as the mean of two biological replicates (*n* = 2) ± SEM. Data were previously checked for their normality, while Tukey’s test was evaluated using OriginPro v.9.1 and MS Excel, for the statistical significance. Then, data was subjected to PCA analysis (see Section 2.14).

### 2.9. Extraction of Plant Material

Aerial parts of *E. elaterium* (P22 and P35) were lyophilized and grounded to a fine powder. The extraction process was performed with ultrasound-assistance (UAE). Briefly, 200 mg of each sample were mixed with solvent at a 1:10 ratio and were treated in an ultrasound bath for 20 min at 30 °C. Each sample underwent three successive extractions: initially with dichloromethane (DCM), followed by methanol (MeOH), and finally with a mixture of methanol and water (MeOH/H_2_O 50:50). The resulting extracts were filtered and evaporated to dryness. All extractions were conducted in triplicate.

### 2.10. Ultra-High-Performance Liquid Chromatography-High-Resolution Mass Spectrometry (UPLC-HRMS) Analysis

For the ultra-high-performance liquid chromatography-high-resolution mass spectrometry (UHPLC-HRMS) and high-resolution tandem mass spectrometry (HRMS/MS) experiments, a Vanquish UHPLC coupled to an Orbitrap Exploris 120 mass spectrometer (both Thermo Fisher Scientific, Waltham, MA, USA) was utilized. Separation was achieved using a Supelco Ascentis Express (Sigma-Aldrich, St. Louis, MO 63103, USA) C18 column (150 mm × 2.1 mm, 2.0 µm) maintained at a stable temperature of 40 °C.

The mobile phase consisted of (A) water (H_2_O) obtained from a Millipore Direct-Q 3 UV purification system (Millipore Sigma, Burlington, MA, USA) containing 0.1% formic ac-id (Optima; Fisher Scientific, Waltham, MA, USA) and (B) acetonitrile (ACN; Supelco, Sigma-Aldrich, St. Louis, MO 63103, USA). The gradient elution program started with 5% B, which was held for 1 min, increased linearly to 100% B over 14 min and was maintained for 2 min. Finally, the system returned to the initial conditions for system equilibration. The total acquisition time was 20 min, with a flow rate of 300 μL/min. The injection volume was 5 μL and the autosampler temperature was set at 7 °C. Samples were prepared at a concentration of 300 μg/mL using a MeOH/H_2_O (90:10, *v*/*v*) solution as dilution solvent.

Mass spectra were acquired in both negative and positive ionization modes using a heated electrospray (HESI) source. The HESI conditions for both modes were as follows: vaporizer temperature was maintained at 350 °C, while the ion transfer tube temperature was set at 325 °C; the source voltage was adjusted to 3.4 kV for negative ion mode and 3.8 kV for positive ion mode. Sheath gas and auxiliary gas were adjusted at 45 and 20 arbitrary units, respectively. Sweep gas was kept at 0 arbitrary units. The HRMS data were acquired in full scan mode over a mass range of 113–1000 *m*/*z*, with a resolving power of 60,000 (full width at half maximum at *m*/*z* 200). HRMS/MS experiments were performed at a resolving power of 30,000 (full width at half maximum at *m*/*z* 200) in “top 3” data-depended acquisition mode by employing normalized collision energy steps of 30%, 50% and 150%. Data acquisition was performed using Xcalibur 4.6. Software and data processing were carried out using Freestyle 1.8 software (Thermo Fisher Scientific Inc., Waltham, MA 02451, USA).

Compound annotation was conducted by examining the base peak (BP) chromatograms of the samples. Peaks were selected using a peak-to-peak analysis and the extracted ion chromatogram (EIC) method, yielding the corresponding full scan spectra. The Freestyle software calculator tool was employed for the proposed elemental composition of each *m*/*z* with error values below 5 ppm, supported by isotopic patterns and ring double-bond equivalent (RDBeq.) values. Metabolite identification was further supported by HRMS/MS spectra and fragmentation patterns, as well as reference to literature data and spectral libraries, including MassBank and NIST 2.4. Additionally, quantitative trends of the identified peaks were monitored across the samples analyzed by comparing the relative peak area intensities obtained from LC-HRMS/MS chromatograms. Peak areas were extracted using consistent parameters across all samples, allowing semi-quantitative comparison of metabolite abundance between conditions.

### 2.11. Cell Lines Bioassays

The SH-SY5Y human neuroblastoma cell line, DU-145 human prostate carcinoma cells, and the MCF-7 (HTB-22™) human breast adenocarcinoma cell line were acquired from ATCC^®^ Manassas, VA, USA). SH-SY5Y cells are commonly utilized as an *in vitro* model for neurodegenerative disorders [43], MCF-7 serves as a model for breast cancer research [44], and DU-145 is used in studies of prostate cancer [45]. Cells were cultured in Dulbecco’s Modified Eagle Medium (DMEM; Thermo Fisher Scientific Inc., Waltham, MA 02451, USA; Cat. No. A1443001), excluding L-glutamine. For SH-SY5Y and DU-145 cells, the medium was supplemented with 10% (*v*/*v*) heat-inactivated fetal bovine serum (FBS; Thermo Fisher Scientific Inc., Waltham, MA 02451, USA;-Cat. No. A5670501) and 100 U/mL penicillin/streptomycin (Thermo Fisher Scientific Inc., Waltham, MA 02451, USA-Cat. No. 15070063). MCF-7 cells were maintained in DMEM supplemented with 10% (*v*/*v*) heat-inactivated FBS, 100 U/mL penicillin/streptomycin, and 10,000 U/mL non-essential amino acids (Sigma-Aldrich™, St. Louis, MO 63103, USA; Cat. No. 12352207). Cell cultures were incubated in a humidified 5% CO_2_ atmosphere at 37°C (CO_2_ Incubator, Model MCO-15AC, SANYO Electric Co., Ltd Osaka, 530-0053, Japan), and the growth medium was refreshed three times per week. For treatments, cells grown in 6-well plates were exposed to varying concentrations of methanolic extracts, which were dissolved in 10 μL dimethyl sulfoxide (DMSO) and diluted in DMEM to final concentrations ranging from 0.1 μg to 1000 μg. Treatments were administered for both 24- and 48 h durations. Control groups received equivalent concentrations of DMSO without extract. Each treatment was conducted in three independent experiments per cell line, using separate cell cultures under identical conditions.

### 2.12. ROS Measurements on Cell Cultures

ROS levels were measured using 10 mM of the oxidant sensitive fluorescent acetyl ester CM-H_2_DCFDA (5-(and-6)-chloromethyl-2′,7′–dichloro-dihydro-fluorescein diacetate—(Thermo Fisher Scientific Inc., Waltham, MA 02451, USA; Cat. no. C6827) dissolved in Dimethyl sulfoxide (DMSO Sigma Aldrich™, St. Louis, MO 63103, USA; Cat. no. 67-68-5). CM-H_2_DCFDA is an oxidative stress indicator that moves through the cell membrane by passive diffusion; CM-H_2_DCFDA is primarily oxidized by hydrogen peroxide and, to a lesser extent, other reactive oxygen species, thus serving as an indicator of overall oxidative stress rather than identifying specific ROS. Inside the cell the ester’s acetate groups are cleaved by intracellular esterases and oxidation by ROS leads to the formation of the fluorescent dichlorofluorescein (DCF) product, which can be detected via fluorometry. The procedure used was as follows: Cells were collected via trypsinization and centrifugation at 1450 rpm for 5 min. Subsequently, samples were incubated continuously for 30 min in the presence of CM-H_2_DCFDA diluted in serum free medium at 37 °C. The ester was subsequently removed, prior to further incubation, for 20 min in serum free medium. Cells were washed three times with PBS buffered solution and centrifuged for 5 min. The obtained supernatant was used for fluorescent measurements in a Versa Fluor Fluorometer System (model 170-2402; Bio-Rad Laboratories, Inc., Hercules, CA 94547, USA). The excitation filter was set at 490 nm and emission at 520 nm. Each set of experiments was performed in duplicates. Total ROS was expressed as fluorescent units/μg of protein extracts [46].

### 2.13. Cell Death Measurements

Cell death was assessed using the Trypan blue exclusion assay following a 48 h incubation period using the extracts [46]. The results were reported as the mean of three independent experimental replicates. Cytotoxicity levels were calculated based on the method described in [47].

### 2.14. Data Preprocessing and Statistical Analysis

For the PCA analysis of biochemical parameters Principal Component Analysis (PCA) was performed using Python v. 3.10.5 (Python Software Foundation, Beaverton, OR 97008-7105, USA) and the scikit-learn library [48] to reduce data dimensionality and identify primary variance components.

## 3. Results

### 3.1. Morphology, Anatomy, and Histochemistry

**Figure 2:**
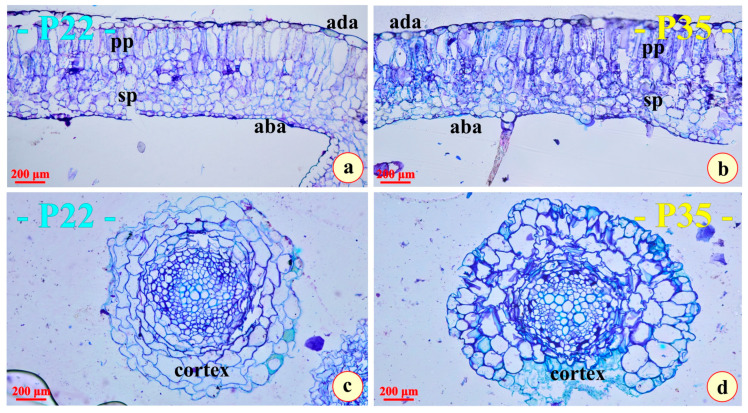
Micrographs of cross-sections from epoxy-embedded leaves and roots stained with toluidine blue “O”. (**a**) Cross-section from a P22 leaf. (**b**) Cross-section from a P35 leaf. (**c**) Cross-section of the primary root from a plant in the P22 group. (**d**) Cross-section of the primary root from a plant in the P35 group. (**c**,**d**) The first xylem elements can be observed, located at the center of the root. Note: ada = adaxial epidermis; pp = palisade parenchyma; sp = spongy parenchyma; aba = abaxial epidermis.

At the end of each of the two 10-day thermal stress experiments, both the plants cultivated at 22 °C (P22) and those cultivated at 35 °C (P35) were dried and weighed to determine their dry mass. As indicated in Table 1, the dry mass of P22 was significantly higher than that of P35. On the contrary, the plant axis diameter measured at the region of the transition zone (diameter of the transition zone was double-measured by a digital caliper (Harley Benton, Bavaria, Germany) and at the microscale during microscopic observations) was to be found higher in P35. Measurements were performed in the transition zone. Microscopic observations of thin sections revealed no significant differences between P22 and P35 (Figure 2a,b).

The cells of the palisade parenchyma are accommodated in a single, distinct layer occupying less than 50% of the width of the mesophyll. A not-always-discernible subjacent second inner layer of the short, palisade-like cells can usually be observed (Figure 2a,b). The spongy parenchyma is very compact and confined to the lower part of the mesophyll, in both leaf types. The mesophyll cells do not seem to accumulate osmiophilic secondary metabolites. Both the upper and the lower epidermal tissues are thin and single-layered. The lower epidermis is composed of significantly smaller cells.

Uncommonly for a xerophyte, none of the epidermal cells seem to accumulate secondary metabolites, since no traits of enclosed osmiophilic materials were observed in the epidermal cells of both leaf types (Figure 2a,b). Both leaf types are amphistomatic. Multicellular, elongated, protective hairs (Figure 2b) as well as capitate secretive trichomes appear on the lower surfaces of the leaves.

The structure of the primary roots does not seem to present any differences (Figure 2c,d). The conductive tissue seems equally developed, while the cortex is composed of parenchyma cells of equal magnitude (Figure 2c,d).

**Figure 3:**
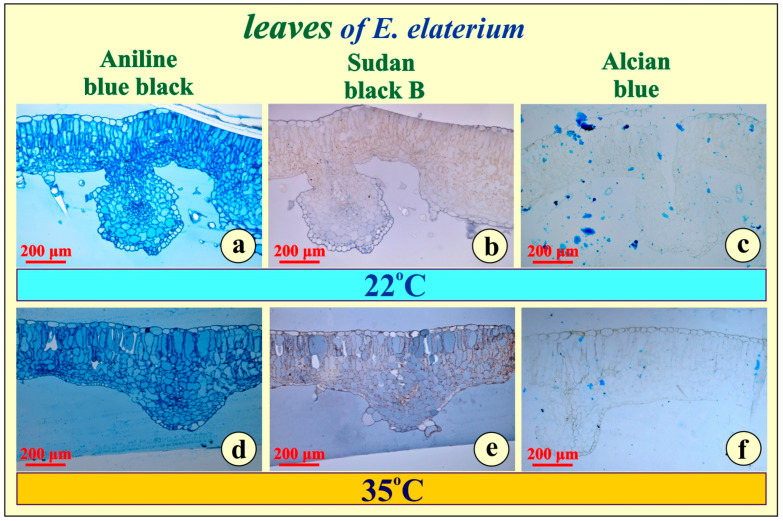
Micrographs of cross-sections from epoxy-embedded leaves after histochemical investigation. Upper row shows leaves from P22: (**a**) Aniline blue black; (**b**) Sudan black B; (**c**) Alcian blue. Lower row shows leaves from P35: (**d**) Aniline blue black; (**e**) Sudan black B; (**f**) Alcian blue.

The histochemical reagents, applied for testing metabolite accumulation in the plastic-embedded leaf tissue, did not give any positive reaction for either of the leaf types in either experiment (Figure 3a–f).

**Figure 4:**
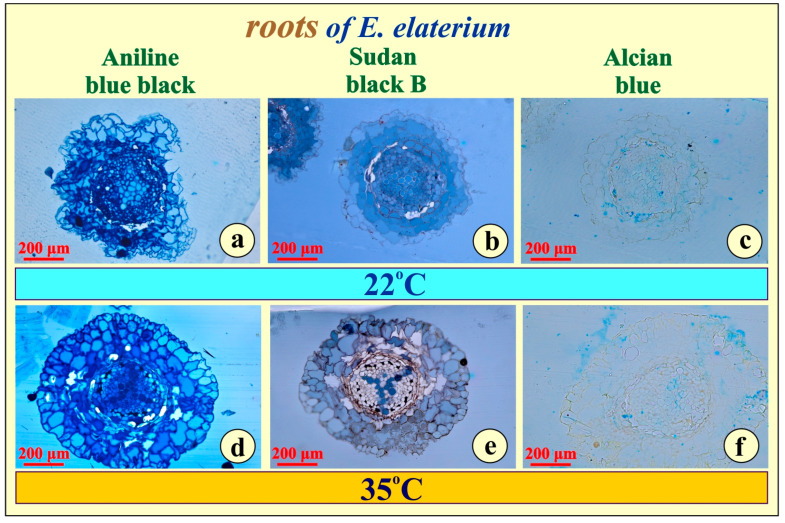
Micrographs of cross-sections from epoxy-embedded primary roots after histochemical investigation. Upper row shows roots from P22: (**a**) Aniline blue black; (**b**) Sudan black B; (**c**) Alcian blue. Lower row shows roots from P35: (**d**) Aniline blue black; (**e**) Sudan black B; (**f**) Alcian blue.

A histochemical investigation was also executed for testing metabolite accumulation in the sections of plastic-embedded root tissue. No proteins (Figure 4a,d), lipids (Figure 4b,e), or polysaccharides (Figure 4c,f) were demonstrated in the two root types for either experiment.

**Figure 5:**
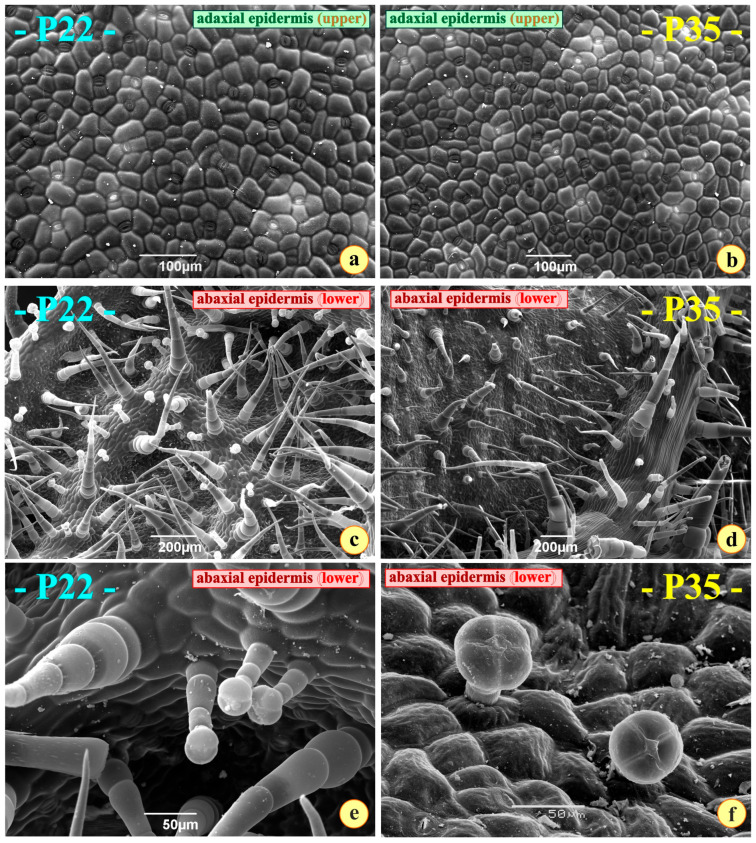
Scanning electron micrographs of the epidermal tissue. (**a**) Upper epidermis from a P22 leaf; (**b**) upper epidermis from a P35 leaf; (**c**) lower epidermis from a P22 leaf; (**d**) lower epidermis from a P35 leaf; (**e**) detail from the lower epidermis of a P22 leaf and the glandular trichomes; (**f**) lower epidermis of a P35 leaf with three glandular trichomes.

Interesting micrographs were obtained after observing the leaf tissues in a scanning electron microscope. The adaxial (upper) surfaces of the two leaf types appear rather similar. They do not possess trichomes (Figure 5a,b). A stomatal density of approximately 220 stomata/mm^2^ was measured on the adaxial epidermis of the P22 leaves; on the P35 leaves, 123 stomata/mm^2^ were counted (Table 2). Guard cells are at a slightly lower level compared to the other epidermal cells. The number of stomata of the abaxial epidermis was impossible to estimate on the P22 leaves due to the dense indumentums. The stomata of both surfaces in both plant groups are of the anomocytic type [10,49] (Figure 5a,b).

The abaxial (lower) surface is loaded with two types of trichomes: elongated, multicellular, uniseriate acute trichomes; shorter, pilate, glandular trichomes (Figure 5c,d). The glandular trichomes end with a head structure composed of four secreting cells (Figure 5e,f).

As demonstrated in Table 2, the number of stomata and the number of glandular trichomes vary in the two plant types. It seems strange that the lower surface of the P35 leaves is practically free of glandular trichomes (Table 2). Only a few of these secreting structures can be observed (Figure 5f).

### 3.2. Physiology

**Figure 6:**
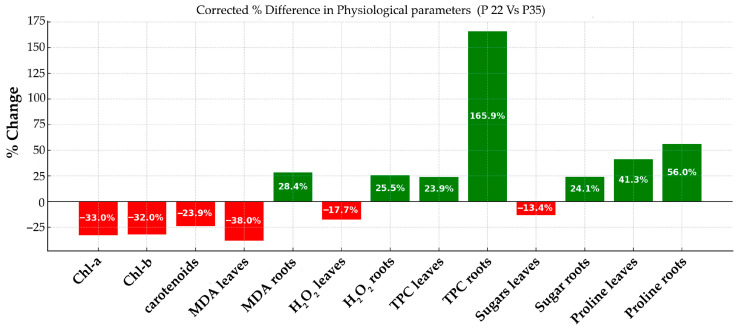
The bar chart titled illustrates the percentage change (positive—green bars; negative—red bars) in various physiological parameters of plants grown at 22 °C (P22) compared to those grown at 35 °C (P35).

Figure 6 reveals that exposure to 35 °C leads to a decrease in photosynthetic pigments and oxidative stress markers in leaves, while roots exhibit increased oxidative stress and strong antioxidant responses. Additionally, soluble sugar and proline accumulation in roots points to a distinct organ-specific stress-mitigation strategy. These findings collectively underscore a thermal-stress-induced physiological reprogramming in plants.

Exposure to elevated temperature (35 °C) resulted in significant physiological alterations in plants compared to those grown at 22 °C. Notably, there was a marked reduction in photosynthetic pigments, with chlorophyll-a, chlorophyll-b, and carotenoids in leaves decreasing by 33.0%, 32.0%, and 23.9%, respectively, indicating impaired photosynthetic efficiency. Oxidative stress responses seem to be organ-specific; malondialdehyde (MDA) and hydrogen peroxide (H_2_O_2_) levels decreased in leaves but increased in roots, suggesting reduced lipid peroxidation in the aerial parts and heightened stress in the root system. Antioxidant activity, as indicated by total phenolic content (TPC), showed a substantial increase, especially in roots (165.9%), reflecting an enhanced protective response. Soluble sugar content decreased in leaves (−13.4%) but rose in roots (+24.1%), pointing to a possible redistribution of carbohydrates under stress. Additionally, proline, a key osmoprotectant, accumulated significantly in both leaves and roots, with increases of 41.3% and 56.0%, respectively, underscoring its role in mitigating thermal stress. Overall, these results highlight a complex, tissue-specific physiological adaptation to high-temperature conditions.

To reduce dimensionality and visualize multi-variation, PCA was conducted separately on biochemical parameters measured in leaves and roots. For each dataset, variables were centered and scaled prior to PCA. The first two principal components (PC1 and PC2) were retained and, together, they explain a substantial proportion of the total variance (see axis labels for exact percentages).

**Figure 7:**
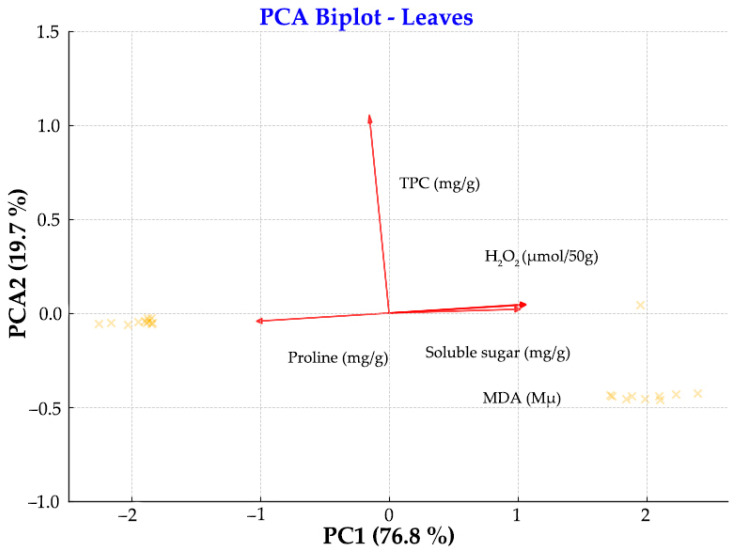
Principal Component Analysis (PCA) biplot of leaves. Biplot displays the first two principal components (PC1 and PC2) in leaves; PC1 primarily reflects variation in MDA and H_2_O_2_, while PC2 captures variation in TPC, sugars, and proline.

In the leaf PCA biplot (Figure 7), PC1 captures variation largely associated with oxidative stress indicators such as MDA and H_2_O_2_, whereas PC2 distinguishes nutrient and metabolite levels such as TPC, sugars, and proline. This vector arrangement indicates that in leaf tissues, proline concentration is inversely related to soluble sugars, H_2_O_2_, and MDA levels along the PC1 (76.8% variance explained). TPC varies independently from the other vectors, primarily contributing to vertical separation along PC2 (19.7% variance explained). The antagonistic relationship between L-proline and the other metabolites along PC1 is an important feature of the data structure. The vector for MDA leaves is oriented distinctly, indicating its strong, unique contribution to PC1. The relative separation and direction of the vectors imply low collinearity between oxidative damage and primary metabolite profiles in leaves. Although MDA and H_2_O_2_ are related markers of oxidative stress, both were included in the PCA to capture different aspects of oxidative damage. The moderate correlation between them does not invalidate the analysis, but their inclusion may influence component loadings. The relative separation and direction of vectors imply low collinearity between oxidative damage and primary metabolite profiles in leaves. In fact, the proline, MDA and soluble sugar are negatively related because the vectors have opposite directions. As for TPC, it is perpendicular to the last vectors, which means that there is no correlation between the last three parameters and the TPC.

**Figure 8:**
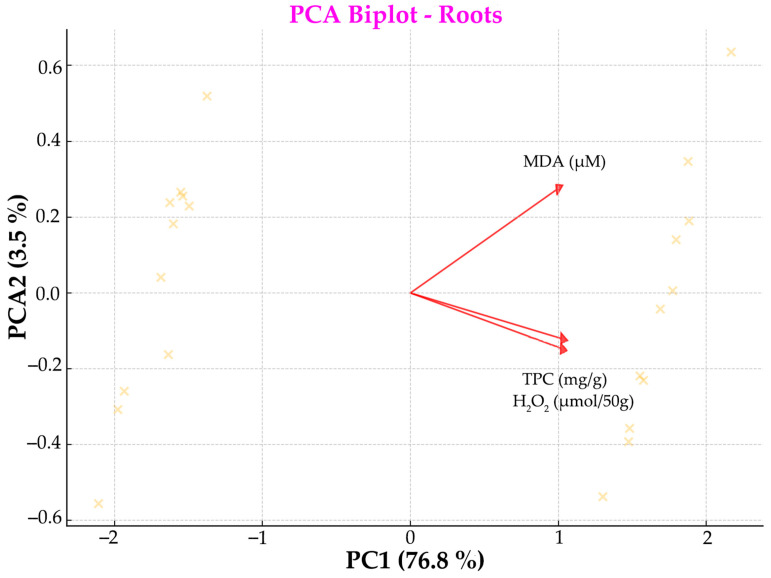
Principal Component Analysis (PCA) biplot of roots. The biplot illustrates the first two principal components in roots. MDA, TPC, and H_2_O_2_ have the same projection on PC1, indicating that they are strongly related, in addition to the fact that the projections on PC2 are opposite because the contribution of PC2 is significantly smaller than that of PC1.

The root PCA biplot (Figure 8) reveals a different pattern. Here, MDA roots and H_2_O_2_ roots load more closely together, suggesting a shared response mechanism in oxidative stress. TPC roots diverges orthogonally, reflecting a distinct contribution to PC2. The tighter clustering of vectors indicates a higher correlation between measured variables in roots compared to leaves. This vector arrangement reveals that, in root tissues, all three measured parameters (MDA, TPC, and H_2_O_2_) tend to increase together along PC1 (76.8% variance explained), suggesting they are positively correlated. However, PC2 (3.5% variance explained) captures the contrasting behavior between MDA (which loads positively) versus TPC and H_2_O_2_ (which load negatively), indicating that, while these parameters generally co-vary, there is some independent variation where MDA can increase while TPC and H_2_O_2_ decrease, or vice versa.

Vector directions represent the influence of each variable on the principal components, while vector lengths approximate the variance explained by each. These biplots thus highlight how stress biomarkers and metabolites vary and co-vary differently in leaves and roots, suggesting organ-specific biochemical responses.

### 3.3. Cytotoxicity Assays

**Figure 9:**
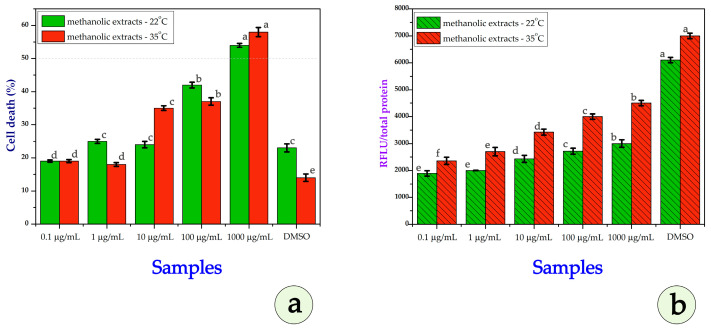
Effects of *E. elaterium* methanolic leaf extracts from P22 (green) and P35 (red) plants on MCF-7 cells: (**a**) cell death (%) across a concentration range (0.1–1000 µg/mL), with the IC_50_ indicated by a horizontal line; (**b**) intracellular ROS levels (relative fluorescence units (RFLUs)/μg protein). Data represent mean ± SD of three replicates (*n =* 3 independent experiments). Statistical differences were assessed using the compact letter display method (CLD; different letters indicate significant differences) and Tukey’s test (*p* ≤ 0.05).

Figure 9a depicts the effect of methanolic extracts in MCF-7 cells. At low concentrations (0.1–1 µg/mL), both extracts cause relatively low cell death (~18–25%), with slightly more cell death from P22 at 1 µg/mL. At 10 µg/mL, both extracts show a marked increase; P35 induces higher cell death (~35%) than P22 (~25%). At 100 µg/mL, the trend reverses, with P22 causing more cell death (~42%) than P35 (~36%). At 1000 µg/mL, both extracts result in high cell death (~54–58%), with P35 being slightly higher. P22 extracts exhibited an IC_50_ of ~71.5 µg/mL, whereas the IC_50_ for P35 could not be reliably determined, suggesting lower cytotoxic potency. IC_50_ values were estimated by fitting a nonlinear regression to the cytotoxicity data (nonlinear regression analysis—four-parameter logistic model; Python, Matplotlib, v. 3.10.5, was implemented). P22 extracts exhibited an IC_50_ of approximately 71.5 µg/mL, whereas the IC_50_ for P35 could not be reliably determined within the tested concentration range, suggesting lower cytotoxic potency. Although cell death in P35 reached 58% at 1000 µg/mL, the nonlinear regression model failed to fit a typical sigmoidal dose–response curve due to the gradual slope and minor variation at low doses. Consequently, the calculated IC_50_ value was unrealistically high and does not reflect the observed biological activity. Based on raw data, the IC_50_ for P35, between 100 and 1000 µg/mL DMSO, was used as a control, while the extracts were diluted in DMSO; minimal cytotoxicity is observed for both conditions, as expected from a positive control. The effect on ROS levels of treatment of MCF-7 cells with methanolic extracts is depicted in Figure 9b. ROS increased with concentration for both extracts. The extract from P35 caused higher ROS levels at intermediate–high concentrations, consistent with increased cytotoxicity. At each concentration, ROS levels are higher with the extract from heat-stressed plants. ROS production is likely dose-dependent. The observed variation in DMSO controls reflects biological variability between independent experiments performed on separately cultured MCF-7 cells. All the treatments, including the controls, were normalized and statistically analyzed within each experiment. Figure 9b shows ROS levels increasing with concentration for both extracts, with P35 causing higher ROS levels at intermediate–high concentrations. At each concentration, ROS levels are higher with P35.

Overall, in MCF-7 cells, both extracts demonstrated a concentration-dependent cytotoxic effect, with P22 generally showing higher potency at mid-to-high concentrations, while P35 induced greater ROS production, particularly at intermediate doses. This suggests that heat stress may enhance oxidative-stress-related pathways without necessarily increasing the overall cytotoxic potency in this cell line.

**Figure 10:**
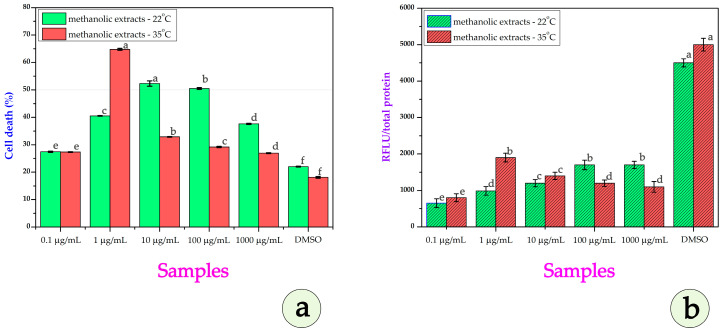
Effects of *E. elaterium* methanolic leaf extracts from P22 (green) and P35 (red) plants on DU-145 cells: (**a**) cell death (%) across a concentration range (0.1–1000 µg/mL), with the IC_50_ indicated by a horizontal line; (**b**) intracellular ROS levels (RFLU/μg protein). Data represent mean ± SD of three replicates (*n* = 3 independent experiments). Statistical differences were assessed using the CLD methodology and Tukey’s test (*p* ≤ 0.05); different letters indicate significant differences.

Examining the data on the cytotoxic effect of methanolic extracts in DU-145 (human prostate cancer cell line), Figure 10a shows a different pattern. At 0.1 µg/mL, both extracts cause similar low cytotoxicity (~27%). At 1 µg/mL, P35 sharply increases cell death (~65%) while P22 reaches ~40%. At 10 µg/mL, P22 shows greater cytotoxicity (~52%) than P35 (~33%). At 100 µg/mL, P22 maintains strong activity (~50%), while P35 declines (~28%). At 1000 µg/mL, P22 still induces ~42% cell death, while P35 plateaus (~26%). In the DMSO control condition for DU-145 cells, baseline cytotoxicity remains low for both extracts, with approximately 22% cell death observed for the 22 °C extract and around 18% for the 35 °C extract. This minimal background effect confirms that the observed cytotoxicity across the tested concentrations is primarily due to the methanolic extracts themselves and not influenced by the solvent, supporting the specificity of the extract-induced cellular responses. The estimated IC_50_ value was approximately 0.91 μg/mL for the P22 extract. In contrast, the IC_50_ for the P35 extract was estimated to be approximately 9.20 μg/mL, indicating lower potency under these conditions.

In the ROS analysis (Figure 10b), at 0.1 µg/mL, both extracts were found to cause similarly low ROS levels. At 1 µg/mL, P35 shows a marked increase, surpassing P22. At 10 µg/mL, both show moderate ROS increases, with the increase for P35 being slightly higher. At 100–1000 µg/mL, P22 produces a greater ROS while P35’s ROS declines, possibly due to the saturation or degradation of its pro-oxidant constituents.

Generally, in DU-145 cells, P35 extract exhibited strong cytotoxicity and ROS induction at very low concentrations, but its effect declined sharply at higher doses, possibly due to compound degradation or cellular adaptation. In contrast, P22 maintained consistently cytotoxic with ROS activity across a wider concentration range, indicating a more stable bioactive profile in this cell type.

**Figure 11:**
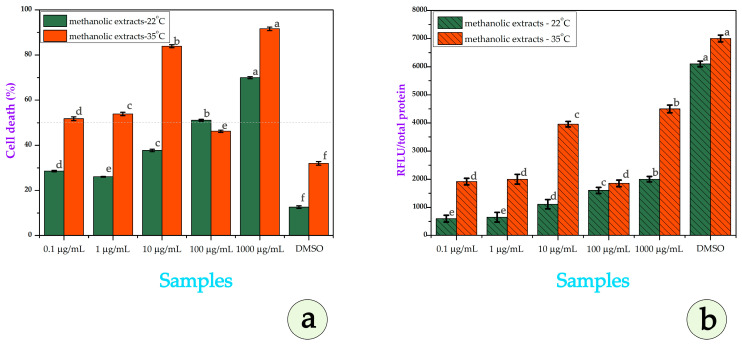
Effects of *E. elaterium* methanolic leaf extracts from P22 (green) and P35 (red) on SH-SY5Y cells: (**a**) cell death (%) across a concentration range (0.1–1000 µg/mL), with the IC_50_ indicated by a horizontal line; (**b**) intracellular ROS levels (RFLU/μg protein). Data represent mean ± SD of three replicates (*n* = 3 independent experiments). Statistical differences were assessed using the CLD methodology and Tukey’s test (*p* ≤ 0.05); different letters indicate significant differences.

In Figure 11a, at 0.1–1 µg/mL, we can see that P35 undergoes higher cytotoxicity (~27–28%) than P22. At 10 µg/mL, P35 shows sharply increased cell death (~84%) versus P22 (~37%). At 100 µg/mL, P22 surpasses P35 (~51% vs. ~47%). At 1000 µg/mL, P35 again shows stronger effects (~91% vs. ~67% for P22). The IC_50_ for P22 is ~155.6 µg/mL; for P35, it is ~1.23 µg/mL, indicating much higher potency after heat stress. Minimal cytotoxicity is observed in the DMSO controls. The calculated IC_50_ value for the P22 extract was approximately 155.6 μg/mL, indicating relatively low cytotoxic potency. In contrast, the P35 extract exhibited a markedly enhanced cytotoxic effect, with an IC_50_ of approximately 1.23 μg/mL, suggesting that exposure to 10-day stress conditions significantly increased the bioactive potential of the plant material. Minimal cytotoxicity is observed in the DMSO positive control, confirming that the measured effects are specific to the extracts, though the extract from P35 maintains slightly elevated background cell death (~31%), potentially due to the residual bioactive compounds. In conclusion, for SH-SY5Y cells, the P35 extract showed markedly greater potency and ROS induction across most concentrations, with a particularly steep increase in cytotoxicity at 10 µg/mL and again at 1000 µg/mL. These findings indicate that heat stress substantially boosts the neurotoxic potential of *E. elaterium* extracts, likely via enhanced pro-oxidant metabolite production.

In our study, ROS levels were evaluated in cell lines following treatment with methanolic extracts derived from P22 and P35, across a range of concentrations. The extracts were dissolved in DMSO; as such, a DMSO-only treatment group was included as the vehicle control. We would like to highlight that DMSO is not always biologically inert. In fact, reports in the literature suggest that DMSO may itself induce oxidative stress or enhance ROS levels in certain cell types, including SH-SY5Y cells [50]. This underscores the importance of including DMSO controls, which we did in our experiments to carefully assess the net ROS impact that can be attributed to the plant extracts alone.

### 3.4. Extraction and LC-HRMS/MS Analyses of Methanol Extracts

The dried material, following pulverization, was sequentially extracted using ultrasound-assisted extraction (UAE) with dichloromethane (DCM), methanol (MeOH), and a mixture of MeOH and H_2_O in a proportion of 50:50, *v*/*v*, yielding 30.0%, 3.90%, and 13.37% (*w*/*w*) for the P22 extracts and 28.93%, 6.27%, and 22.97% (*w*/*w*) for the P35 extracts, respectively.

The methanol extracts of the P35 and P22 samples, which exhibited cytotoxic activity against MCF-7, SH-SY5Y, and DU-145 cancer cell lines, were subjected to UHPLC-HRMS/MS analysis, aiming to identify the metabolites that are potentially responsible for the observed bioactivity. Chromatographic and spectrometric data for all tentatively identified metabolites are summarized in Table 3.

The majority of the compounds were annotated based on high-resolution tandem mass spectrometry (HRMS/MS) data acquired in negative ionization mode, as this ionization mode yielded a more informative and richer profile. Positive ion mode data were selectively used for compounds that were poorly ionized in negative ion mode, thereby ensuring comprehensive chemical characterization.

LC-HRMS and HRMS/MS analyses revealed that both extracts (P22 and P35) exhibited qualitatively similar profiles, comprising a diverse array of secondary metabolites, including triterpenoids and their glycosylated derivatives, flavonoid glycosides, and various lipid classes such as sphingolipids, glycerolipids, glycerophospholipids, and glycolipids (Table 3). While the qualitative profiles were largely conserved between the two samples (P22 and P35), clear quantitative differences were observed, consistent with the variations observed in the methanolic extraction yields.

**Figure 12:**
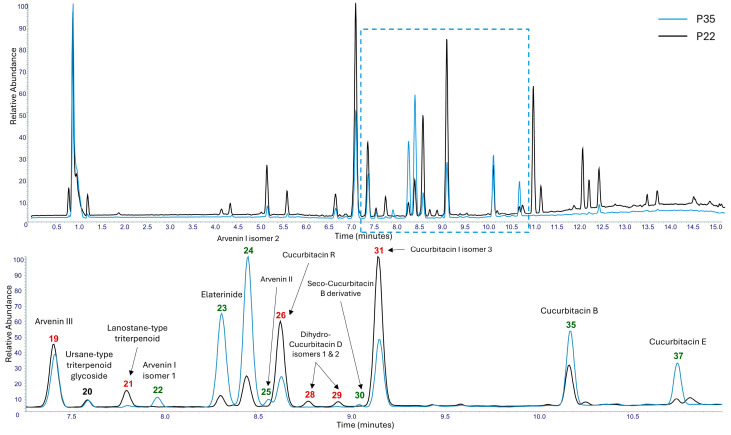
Overlaid base peak (BP) chromatograms from LC–HRMS analysis of the P22 (black) and P35 (blue) methanolic extracts of *Ecballium elaterium* in negative ion mode. The region of the chromatogram where major differences are observed is highlighted with a square. Top: zoomed-in view of the highlighted region, illustrating key differences in peak intensity and distribution between the two extracts. Bottom: ID numbers of the annotated compounds are shown in green (more abundant in P35), red (less abundant in P35), and black (similar abundance in both extracts).

A total of 49 secondary metabolites were detected, of which 38 were tentatively annotated. Among them, as shown in the base peak (BP) chromatogram acquired in negative ion mode (Figure 12), the major constituents of the P22 extract included three Cucurbitacins: Cucurbitacin I 2-O-glucopyranoside ([M+FA-H]^−^ at *m*/*z* 721.3434, 7.15 min), Cucurbitacin I isomer 3 ([M+FA-H]^−^ at *m*/*z* 559.2906, 9.16 min), and Cucurbitacin R ([M+FA-H]^−^ at *m*/*z* 561.3063, 8.64 min). There were also a dissacharide, trehalose ([M+Cl]^−^ at *m*/*z* 377.0850, 0.92 min), and a glycerolipid, Gingerglycolipid A ([M+FA-H]^−^ at *m*/*z* 721.3643, 11.07 min). In contrast, the P35 sample was dominated by different Cucurbitacins: Arvenin I ([M+FA-H]^−^ at *m*/*z* 765.3696, 8.45 min), a 2-O-glucopyranoside of Cucurbitacin B; Elaterinide ([M+FA-H]^−^ at *m*/*z* 763.3542, 8.31 min), a 2-O-glucopyranoside of Cucurbitacin E; and Cucurbitacin B ([M+FA-H]^−^ at *m*/*z* 603.3168, 10.18 min). Notably, Cucurbitacin I 2-O-glucopyranoside ([M+FA-H]^−^ at *m*/*z* 721.3434, 7.15 min) and trehalose ([M+Cl]^−^ at *m*/*z* 377.0850, 0.92 min) were also among the major compounds in the P35 extract.

In the positive ion mode, the predominant compounds in both extracts were sphingolipids, which produced intense signals that, in some cases, suppressed the ionization of other analytes. For this reason, the BPC from negative ion mode was selected for visualization (Figure 12), as it provided greater chemical diversity and was more informative for compound annotation.

HRMS/MS fragmentation analysis confirmed the structural characteristics of multiple Cucurbitacin-type triterpenoids and their corresponding glycosides beyond the major compounds reported above. Several Cucurbitacins were detected, including Cucurbitacins E ([M+FA-H]^−^ at *m*/*z* 601.3015, 10.76 min), R ([M+FA-H]^−^ at *m*/*z* 561.3063, 8.64 min), and S ([M+ H]^+^ at *m*/*z* 499.3045, 7.17 min). Cucurbitacin I was detected in two isomeric forms ([M+ H]^+^ at *m*/*z* 515.3000, 6.70 min, and [M+FA-H]^−^ at *m*/*z* 559.2906, 9.16 min). Also, dihydro-Cucurbitacin D was detected in two isomeric forms ([M+FA-H]^−^ at *m*/*z* 561.3063, 8.78 and 8.94 min). Interestingly, Cucurbitacin B was also present in a seco-form ([M-H]^−^ at *m*/*z* 589.3016, 9.05 min). The corresponding glycosides were particularly abundant in the P35 extract, including the 2-O-glucopyranoside of Cucurbitacin J, alongside the major glycosylated forms of Cucurbitacins B (Arvenin I), E, and I. Arvenin II ([M+FA-H]^−^ at *m*/*z* 561.3063, 8.58 min) and III ([M+FA-H]^−^ at *m*/*z* 723.3588, 7.41 min) were also detected alongside Arvenin I, as well as less common Cucurbitacin derivatives such as Khekadaengoside K ([M-H]^−^ at *m*/*z* 561.2701, 6.68 min) and Deoxocucurbitoside B ([M+FA-H]^−^ at *m*/*z* 835.4116, 8.75 min). The presence and relative abundance of these compounds suggest a prominent role for Cucurbitacins—especially their glycosides—under stress conditions and in the observed bioactivity of the extracts.

Quantitative assessments based on peak area comparisons between P22 and P35 extracts revealed a general trend of reduced metabolite abundance in the P35 sample. However, several notable exceptions were observed. Specifically, Cucurbitacins B ([M+FA-H]^−^ at *m*/*z* 603.3168, 10.18 min), E ([M+FA-H]^−^ at *m*/*z* 601.3015, 10.76 min), and S ([M+ H]^+^ at *m*/*z* 499.3045, 7.17 min), as well as the two isomeric forms of Dihydro-Cucurbitacin D ([M+FA-H]^−^ at *m*/*z* 561.3063, 8.78 and 8.94 min) and a Seco-Cucurbitacin B derivative ([M-H]^−^ at *m*/*z* 589.3016, 9.05 min), were found at higher levels in the heat-exposed methanolic extract (P35). In addition, several Cucurbitacin glycosides—including Arvenin I ([M+FA-H]^−^ at *m*/*z* 765.3696, 8.45 min), an Arvenin I isomer ([M+FA-H]^−^ at *m*/*z* 765.3698, 7.97 min), Arvenin II ([M+FA-H]^−^ at *m*/*z* 561.3063, 8.58 min), and Elaterinide ([M+FA-H]^−^ at *m*/*z* 763.3542, 8.31 min)—were also more abundant in the P35 extract. This pattern extended to non-triterpenoid metabolites as well, with higher levels of the disaccharide trehalose ([M+Cl]^−^ at *m*/*z* 377.0850, 0.92 min); the glycerolipids monopalmitin ([M+H]^+^ at *m*/*z* 331.2841, 15.87 min) and monostearin ([M+H]^+^ at *m*/*z* 359.3155, 17.19 min) were also detected to be higher in P35 compared to P22.

These findings suggest that specific metabolite classes, particularly Cucurbitacins and glycerolipids, may be upregulated in response to stress and potentially linked to the observed cytotoxic effects.

## 4. Discussion

### 4.1. Morphology

Most of the morphological and anatomical observations indicate that there are no major differences between the two plant types, although the dry mass of P35 (1.7 ± 0.3 g) is significantly lower compared to that of P22 (2.8 ± 0.4 g). This difference can be attributed to the higher temperature the plants in the P35 group were exposed to. This probably indicates a suppression of the metabolism of these plants, common to most plants under heat stress; since dry mass accumulation reflects the integrated photosynthesis rate and because the combination of drought and heat stress adversely affects photosynthesis, overall plant dry mass is consequently reduced [58]. On the other hand, a rather difficult to explain increase in the diameter of the transition zone of the P35—8.26 ± 0.56 mm^2^ vs. 6.96 ± 0.22 mm^2^ for the P22 was observed. The transition zone (TZ) is considered to be the site for hormonal/signal crosstalk. In addition to regulating root development, it is also implicated in the perception and response to diverse environmental stressors [59]. So, our data with augmented diameter in the TZ, following heat stress, could reveal a possible protection role of the TZ while it accumulates molecules facing stress; it has been demonstrated that, in case of water stress, the ABA protects the transition zone [60,61]. Thus, the increased diameter of the TZ could be attributed to the development of the conductive and mechanical tissue produced as a response to the combination of high temperature and the desiccation conditions this environmental factor imposes.

Another interesting feature and a major difference between the two leaf types has to do with the stomata and the hair cover. Both leaf types are amphistomatic. This is common in most xerophytes. Having stomata on both sides facilitates the rapid gas circulation within the leaf and the exchange during the short periods of moderate environmental conditions that favor photosynthesis [62,63,64]. Although the stomatal frequency could not be counted on the lower epidermis of the P22 leaves due to the heavy pubescence, the difference of the stomatal number per millimeter on the upper epidermis for the two leaf types was significant. It seems that the P35 develop a kind of resistance to water loss by reducing the number of stomata (119 ± 9 stomata/mm^2^); this number is lower in comparison with the number of stomata for the P22, which is almost double (221 ± 28 stomata/mm^2^) [65]. This reduction in stomatal density in P35 likely represents a developmental adjustment to limit transpirational water loss under higher growth temperatures. While amphistomaty generally supports high gas exchange and photosynthetic rates, the lower stomatal density in P35 leaves could improve water conservation during heat stress at the cost of reduced maximum carbon assimilation. Furthermore, it is documented that water stress also results in stomatal closure and thus reduced stomatal conductance [66] and adversely affects photosynthesis due to reduced CO_2_ intake [67]. Finally, because RH can affect stomatal development and leaf anatomy, the fixed difference in RH between chambers represents a potential confounding factor. While temperature was the primary variable of interest and RH was stable within chambers over time, we acknowledge that RH possibly contributed to the magnitude of anatomical differences observed.

It is also very strange that the number of glandular capitate trichomes, present only on the abaxial surfaces of the leaves [68], is drastically reduced in the P35 (see Table 2). Bearing in mind the anti-respiratory effect of the oil produced by these trichomes [69], we may assume that the decrease in the stomatal frequency one of the reactions of the plants in the P35 group against the high temperature. Glandular trichomes play important roles in producing protective secretions, reflecting sunlight and deterring herbivores. Their scarcity in P35 suggests that high-temperature growth may suppress their development, potentially compromising chemical and microclimatic defenses while still retaining some mechanical protection from the elongated trichomes. Heat stress has been reported to cause leaf senescence and decreased leaf area index in maize [70] and tomato [71]. It also has a negative effect on photosynthetic performance [72]. It was also reported that heat stress significantly increases stomatal length [72]. Investigating the leaf anatomy of different cultivars of *Rhododendron hybridum* exposed to 38 °C (heat stress) and comparing their leaf structures to those of the control plants (25 °C) revealed that exposure to heat stress for 6 days increased the thickness of spongy parenchyma, main veins, and upper epidermis, in various degrees, depending on the cultivar [72]. This suggests that heat-stress-adaptive strategies at the anatomical level vary among cultivars. It was also reported that the minor vein area and stomatal density decreased in rice under heat stress (38/28 °C) compared to control plants (30/28 °C) [73]. All the above findings suggest that the plasticity of the leaf anatomy and vein density cannot be attributed to increased photosynthetic rate or stomatal conductance under heat stress.

Following another investigation [74], it was reported that heat stress (40 °C) for 7 days decreased petiole length by 19.4% and 16.88% in WS-6 (heat-sensitive genotype) and WS-1 (heat-tolerant genotype), respectively, which are two *Brassica campestris* L. genotypes. Moreover, the boundary between the spongy and palisade tissues blurred, and the paradermal sections revealed a significant reduction from the upper to the lower epidermis. Heat stress increased the thickness of the leaf lamina, the palisade, and the spongy parenchyma, in various degrees among the cultivars. It also decreased the thickness of the palisade mesophyll and upper epidermis in various degrees as well, in other cultivars, without causing any significant changes in the leaf thickness or the lower epidermis; all the effects of the heat stress mentioned above are attributed to cultivated plants and their varieties.

The primary root structure appeared to be consistent between P22 and P35, with equally developed conductive tissues and similarly sized parenchyma cells in the cortex. No qualitative anatomical differences were noted, suggesting that root structure is relatively stable and less sensitive to variation in growth temperature. This stability may reflect the roots’ critical role in water and nutrient uptake, where maintaining structural integrity is essential regardless of the environmental temperature conditions experienced during growth; temperature fluctuations in the soil are far less pronounced than those above ground.

Concerning differences of leaf and root structure, when compared between the two plant types, it seems negligible. The leaves and the roots are similarly developed, while secondary metabolites common in water or temperature stressed plants are practically absent. Furthermore, the absence of storage compounds (Figure 3 and Figure 4), probably excluding the tannins, which are metabolically inactive end-products, may indicate that, under severe stress, these plants rapidly utilize any reserves for immediate metabolic needs rather than storing them. Alternatively, it may reflect an inherent physiological strategy in which these species maintain minimal storage in vegetative tissues, relying instead on continuous photosynthesis and resource transport for survival.

Concluding our approach to heat stress, we may say that—although heat stress was reported to increase leaf thickness, close some of the stomata, and decrease leaf area index, vein area, and stomatal density (the reactions needed to overcome water loss through transpiration)—the plant responses are genotype-specific. Wild plants, like *Ecballium elaterium*, in which only minor structural differences were detected after exposure to high temperatures, are adapted to hot and arid environments by exploiting various strategies, sometimes far from structural alterations. It should be noted that, under the balanced conditions of water availability, temperature, and light, leaf anatomy itself—particularly the thickness and differentiation of palisade versus spongy parenchyma—is a key determinant of photosynthetic performance. In contrast, under field conditions, plants often experience combined stressors (e.g., heat, drought, and high irradiance), where the interaction between anatomical traits and environmental limitations (such as stomatal closure) further shapes photosynthetic efficiency. This distinction is important for interpreting our results in both controlled and natural contexts.

Anatomical observations suggest that plants grown at higher temperatures (P35) adjust their epidermal traits toward water conservation, as indicated by reduced stomatal density, but at the potential cost of diminished chemical and trichome-based protective capacity. In contrast, plants in the P22 group maintain higher stomatal densities and abundant glandular trichomes, which may support higher photosynthetic rates and stronger defense capabilities under more moderate growth conditions. Overall, growth temperature appears to influence external protective and regulatory features far more than internal leaf or root anatomy. It should be noted that under, balanced conditions of water availability, temperature, and light, leaf anatomy itself—particularly the thickness and differentiation between palisade and spongy parenchyma—is a key determinant of photosynthetic performance. In contrast, under field conditions, plants often experience combined stressors (e.g., heat, drought, and high irradiance), where the interaction between anatomical traits and environmental limitations (such as stomatal closure) further shapes photosynthetic efficiency. This distinction is important for interpreting our results in both controlled and natural contexts.

### 4.2. On the Plant Function

The PCA analyses of P22 and P35 *Ecballium elaterium* provide compelling insights into the distinct biochemical strategies employed by different plant organs in response to elevated temperature. These analyses reveal a clear divergence between leaf and root tissues in how oxidative and metabolic stress signals are integrated, interpreted, and mitigated under heat stress conditions. In leaves, PC1 (Figure 7) predominantly captured oxidative stress markers, with MDA and H_2_O_2_ exhibiting strong contributions; particularly MDA, which displayed a uniquely oriented vector indicative of its distinct role in the oxidative stress response. In contrast, PC2 (Figure 7) was defined by total phenolic content (TPC), soluble sugars, and proline, indicating that antioxidant and osmoprotective mechanisms were regulated independently of lipid peroxidation and H_2_O_2_ levels. The low collinearity between vectors supports the interpretation that multiple, parallel biochemical pathways are engaged under heat stress, reflecting a complex and compartmentalized response.

Indeed, L-proline functions as an osmolyte, keeping the redox equilibrium, via its role as a ROS scavenger [75,76,77]. Hydrogen peroxide (H_2_O_2_) is one of the major reactive oxygen species (ROS) and is therefore included among the targets of ROS scavenging systems [78]. In Figure 6, it can be observed that MDA and H_2_O_2_ exhibit the same tendency; MDA and H_2_O_2_ concentrations in leaves are reduced (38% and 17%, respectively). At first glance, this might suggest a reduction in oxidative stress. However, we interpret these reductions not as an absence of stress but rather as the result of the active and effective engagement of ROS-scavenging pathways. For instance, L-proline levels increased markedly (by 41.3%), which is consistent with its well-documented role as an osmoprotectant and ROS scavenger that stabilizes proteins and membranes and regulates cellular redox status [75,76,77]. In parallel, TPC increased by 23.9%, further supporting enhanced antioxidant activity. It is well documented that heat stress mainly retards photosynthesis and the subsequent reduced photosynthetic pigment production [79] in many plants such as wheat, maize, chickpeas, tomato, and *Medicago sativa* [80,81,82,83,84]. Heat stress is responsible for the triggering of a retrograde signaling pathway in chloroplasts mediated by ROS and H_2_O_2_ [85], leading to both protective and repressive metabolic adjustments. On the other hand, carotenoids, using their conjugated double bonds, functioning as nonenzymatic antioxidants, could also quench the ROS and remove the free radicals [86]. In our data, decreased carotenoids after heat stress may reflect their consumption during photoprotection; such a finding can be seen to be in accordance with [87], where the authors suggested that the decreased levels of carotenoids could be associated with thermal energy dissipation, underlying an adaptive response to abiotic stress. Soluble sugars are involved in abiotic stress responses due to their functions as osmoprotectant and metabolite signaling molecules [80]. Furthermore, they are involved in and feed NADPH-producing metabolic pathways contributing to ROS scavenging [88]. Moreover, soluble sugars also decreased, which may be attributable to suppressed photosynthetic activity and carbon assimilation, as well as to their utilization in stress response metabolic pathways [88,89,90]. Phenolic compounds are also implicated to ROS scavenging, following plant’s oxidative stress [91,92,93,94]. The 23.9% increase in leaves is also in accordance with the tendency of L-proline and soluble sugars, indicating the activation of scavenging mechanisms.

In contrast to the multifaceted response observed in leaves, the root tissue exhibited a more integrated and tightly coordinated biochemical response to heat stress. The root PCA revealed a more integrated response. MDA and H_2_O_2_ vectors were closely aligned, suggesting a shared variance and a co-regulated oxidative stress mechanism in roots; this clustering suggests that ROS accumulation and lipid peroxidation in roots are not independent phenomena but occur in a more synchronized manner. TPC, however, contributed orthogonally to PC2, indicating a unique metabolic response distinct from oxidative damage (Figure 8); this is a distinct role for the antioxidant phenolics in roots, possibly acting independently of the oxidative damage trajectory. The overall tighter clustering of vectors in the root PCA implies a higher degree of correlation among biochemical parameters, reflecting a more unified physiological adaptation to heat stress in roots. Proline does not make a distinct contribution to either principal component, suggesting its influence was less pronounced there, and soluble sugars were not highlighted as major contributors to either PC1 or PC2, suggesting a lesser role or lower variation in the root response. In roots, MDA and H_2_O_2_ were still important, clustering together and contributing strongly to the variation along PC1—though less distinctly than in leaves. The 28.4% increase in MDA in roots indicates elevated lipid peroxidation, a marker of oxidative stress; roots do experience oxidative stress at 35 °C, but to a lesser extent than leaves. The same motif is true for the H_2_O_2_ concentration. The 25.5% increase underlines enhanced reactive oxygen species (ROS) accumulation, but again, roots seem to manage this rise without severe damage (Figure 6). Soluble sugars in roots increased 24.1%, possibly reflecting a metabolic adaptation to osmotic and oxidative stress, aiming to aid cellular protection and energy supply. TPC exhibited the strong activation of antioxidant defenses in roots under heat stress. This pattern suggests that roots activate a coordinated protective mechanism involving antioxidants and osmoprotectants to mitigate heat-induced damage. Overall, the tighter clustering of vectors in the root PCA implies greater correlation among the measured biochemical variables, which we interpret as a more unified, perhaps more stable physiological response to heat stress. Unlike leaves, which showed some suppressed parameters (e.g., chlorophylls, sugars, MDA), roots seem more resilient, with overall upregulation of defense and metabolic markers, effectively maintaining homeostasis without severe damage, in contrast to the more complex and differentiated response seen in the aerial parts of the plant. This could reflect the root’s sheltered position within the soil matrix, as well as its inherently different metabolic demands compared to the leaf.

The implementation of our findings supports the notion that leaves and roots employ organ-specific strategies to manage heat stress, shaped by their distinct physiological roles and environmental exposures. Leaves seem to suffer more stress than roots, showing more pronounced and compartmentalized biochemical disruption, likely due to the direct impact of stress on the photosynthetic machinery and energy metabolism. In leaves, MDA showed a distinct vector orientation and dominated PC1, suggesting a strong and unique oxidative stress response. Furthermore, the low collinearity between MDA and H_2_O_2_ and TPC, sugars, and proline suggests that multiple, independent biochemical pathways were engaged; these data could imply more complex and potentially more intense effects of stress. On the other hand, in roots, MDA and H_2_O_2_ clustered more closely, indicating a more coordinated and less differentiated oxidative stress response. Additionally, our histological results as evidence of mild rather than severe osmotic stress, with L-proline likely serving as the primary osmoprotectant under our experimental conditions, which may have buffered against more drastic cell wall remodeling. The tighter clustering of vectors overall suggests less variation across biochemical responses, which could imply a more controlled or buffered reaction to heat stress. These organ-specific signatures, as revealed by PCA and biochemical analyses, underscore the importance of analyzing stress responses in a tissue-specific context and support the need for targeted approaches in stress physiology research.

### 4.3. Bioassays and LC-HRMS Analysis

The aim of our bioassays was to assess whether the secondary metabolites in the plant’s leaves, produced during thermal stress, may exert cytotoxic effects on cell lines *in vitro*. In order to evaluate the cytotoxic activity of methanolic extracts from *E. elaterium*, we selected three human cancer cell lines representing distinct tissues and clinical challenges: DU-145 (prostate), SH-SY5Y (neuroblastoma), and MCF-7 (breast). The DU-145 cell line models prostate cancer, the second most frequently diagnosed malignancy in men, with approximately 1.5 million new cases reported annually worldwide [94]. The MCF-7 line is a well-characterized estrogen-receptor-positive model of breast cancer—the leading malignant tumor affecting women globally, with an estimated 2.3 million new diagnoses and 630,000 deaths per year across 185 countries [95]. Finally, SH-SY5Y cells represent neuroblastoma, the most prevalent extracranial solid tumor in children, responsible for approximately one in six pediatric-cancer-related deaths [96,97]. Together, these cell lines allow us to assess the broad-spectrum cytotoxic and oxidative-stress-inducing properties of stress-modified plant extracts across cancer types of distinct origin, age prevalence, and biological behavior. Furthermore, we have to underline that, while several studies have analysed the effects of extracts from the juice and seeds of the plant, very few have focused on the activity of its leaf extracts. Moreover, with regard to the SH-SY5Y and DU-145 cell lines, there is no *in vitro* testing using extracts from any part of the plant. Only for the MCF-7 cell lines have previous studies been conducted using *E. elaterium* extracts. However, these extracts were derived from the fruit juice of the plant [18,98]. These studies reported apoptosis and cell death in the MCF-7 cell line upon treatment with extract concentrations of 1 mg/mL and 10 mg/mL.

In our study, the cytotoxic responses to methanolic extracts from *Ecballium elaterium* varied notably across the three cancer cell lines tested. In DU-145 prostate cancer cells, the extract from P35 exhibited stronger cytotoxicity at low concentrations (1 μg/mL), while the extract from P22 proved more effective at higher doses (≥10 μg/mL), suggesting a dose-dependent shift in efficacy (Figure 10a). In contrast, MCF-7 breast cancer cells were more sensitive to the extract from P22 at all concentrations, indicating that the stress-induced phytochemical profile may be less effective or less selectively cytotoxic in this cell type (Figure 9a). It is possible that stress might reduce bioactive compound effectiveness or shift them toward less effective metabolites. Notably, SH-SY5Y neuroblastoma cells responded with pronounced, dose-dependent cell death following treatment with extract from P35 (Figure 11a), highlighting a potent neurotoxic or selective anti-neuroblastoma effect, likely driven by compounds synthesized under heat stress conditions. These cell-type-specific responses underscore the importance of both extract origin and cancer context in evaluating plant-derived anticancer agents. These findings, along with IC50 values, suggest that the bioactivity of plant-derived compounds may vary significantly depending on both the environmental growth conditions and the specific cancer cell line tested. In our experiments, the DMSO control demonstrated both elevated ROS and moderate cell death, consistent with reports that low DMSO concentration can induce mild oxidative stress in mammalian cell lines [99,100]. Interestingly, when extracts were combined with DMSO, cell death increased while ROS levels decreased. A plausible explanation is that antioxidant phytochemicals within the extracts (such as polyphenols and flavonoids) scavenged ROS that was generated by DMSO exposure, thereby lowering measurable ROS levels; at the same time, other bioactive constituents triggered pro-apoptotic or cytotoxic pathways, independently of oxidative stress. Such biphasic effects—simultaneous antioxidant and cytotoxic activities—have been widely reported for plant-derived compounds and purified phenolics, often involving mechanisms such as caspase activation, mitochondrial dysfunction, or cell cycle regulation [101,102,103,104]. Thus, while the precise mechanisms remain to be clarified, our findings suggest that the cytotoxic action of the extracts may proceed, at least in part, through ROS-independent pathways.

These findings suggest that thermal stress alters the phytochemical composition of *E. elaterium* in a manner that modulates its cytotoxic profile across different cancer types. While stress appears to enhance activity against neuronal-derived cells, it may reduce effectiveness in epithelial cancer models such as MCF-7.

LC-HRMS/MS analysis revealed that the predominant constituents in both methanolic extracts (P22 and P35) were Cucurbitacins and their glycosides, comprising 22 of the 38 tentatively annotated compounds. Cucurbitacins are highly oxygenated tetracyclic triterpenoids, widely distributed in plants of the Cucurbitaceae family, including *Ecballium elaterium* [21]. Their primary biological role is defensive, deterring herbivores through their pronounced bitterness [21]. Beyond this, Cucurbitacins have attracted substantial pharmacological interest due to their potent cytotoxic and antiproliferative effects against various cancer types [98,99,105]. Among them, Cucurbitacins B and D are particularly well known for their strong cytotoxicity across multiple cancer cell lines [70]. Over the years, a wide range of Cucurbitacins have been isolated from natural sources, and various semisynthetic derivatives have been developed to optimize their anticancer properties by modifying their core scaffold [70]. In our study, extracts from plants in both the P22 and P35 groups demonstrated significant cytotoxicity against SH-SY5Y (neuroblastoma), DU-145 (prostate), and MCF-7 (breast) cancer cell lines. These findings are in agreement with previous reports of antiproliferative and cytotoxic activity from fruit juice and chloroform or *n*-hexane extracts, as well as from isolated Cucurbitacins derived from *E. elaterium* [18,106,107]. However, to our knowledge, this is the first report demonstrating the cytotoxic activity of methanolic extracts of aerial parts of *E. elaterium* against the MCF-7, SH-SY5Y, and DU-145 cancer cell lines.

**Figure 13:**
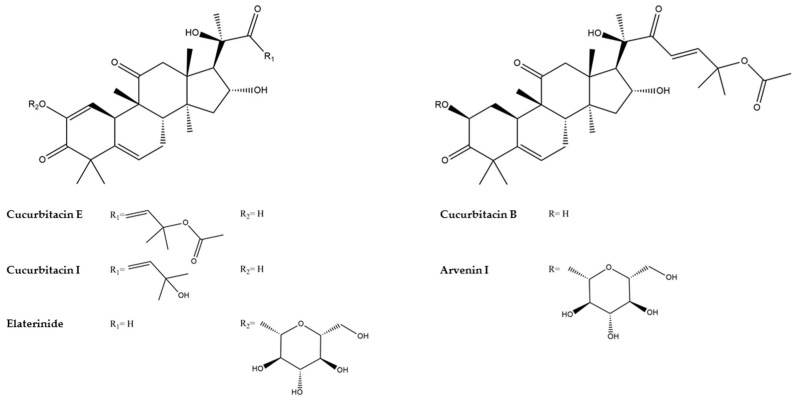
Representative chemical structures of the predominant Cucurbitacins annotated via LC–HRMS/MS in the P22 and P35 extracts.

In P22, the most abundant Cucurbitacins were Cucurbitacin I and its 2-O-glucopyranoside. In contrast, P35 was enriched in Cucurbitacin I 2-O-glucopyranoside, Elaterinide (Cucurbitacin E 2-O-glucopyranoside), and Arvenin I (Cucurbitacin B 2-O-glucopyranoside) (Figure 13). The increased abundance of glycosylated Cucurbitacins in P35 suggests that heat exposure may favor glycoside formation or preservation.

Cucurbitacin I has been shown to exert antiproliferative and cytotoxic activity against a range of cancer types, including breast (MCF-7) cells [18,21,108]. It also acts by inhibiting STAT3 signaling, which is constitutively active in prostate cancer cells [109]. Given its presence in both extracts, this compound likely contributes to the observed cytotoxicity across all three tested cell lines.

Cucurbitacin B, found at significantly higher levels in P35, is among the most studied Cucurbitacins for its anticancer potential. It has been demonstrated to reduce viability in various breast cancer cell lines (MDA-MB-231, SKBR3, MCF-7, 4T1) in a dose- and time-dependent manner (IC_50_ = 18–50 nM) [110]. Mechanistic studies show that Cucurbitacin B induces G_2_/M arrest, apoptosis, and autophagy in MCF-7 cells [111,112,113] and promotes apoptosis and cell cycle arrest in SH-SY5Y cells [107,114]. Recent data confirm its selective cytotoxicity toward cancer cells, including DU-145 [115].

Cucurbitacin E, also more abundant in P35, has exhibited strong cytotoxicity against prostate cancer cells (IC_50_ = 7–50 nM) [115] and MCF-7 cells [116]. Its glycosylated form, Elaterinide, a major constituent of P35, likely contributes to the enhanced cytotoxic profile of this extract.

Although most studies focus on Cucurbitacin aglycones, emerging evidence supports the biological activity of their glycosides. However, existing evidence supports their bioactivity. Arvenin I showed moderate cytotoxicity against MCF-7 cells [117], and in vivo, it enhanced the efficacy of cancer immunotherapy in mice by acting as a covalent kinase activator [57]. Elaterinide and Arvenin I, when tested as a mixture, inhibited the proliferation of both MCF-7 and MDA-MB-231 breast cancer cell lines [118,119]. These findings underscore the potential contribution of glycosylated Cucurbitacins to the cytotoxic effects observed in the P35 extract.

For future studies, targeted isolation of the metabolites that are found to be upregulated under stress conditions should be prioritized. Isolation of these compounds will enable more confident structural elucidation, including validation with authentic standards when available, and direct evaluation of their cytotoxic activity against cancer cell lines, providing clearer insights into their individual bioactivities. Additionally, testing isolated compounds both individually and in combination will enable the assessment of potential synergistic or antagonistic interactions, which is essential for understanding the overall therapeutic potential of *Ecballium* extracts.

In summary, the higher cytotoxic activity of the P35 extract against DU-145 prostate cancer cells can be attributed to its elevated levels of Cucurbitacin B, Cucurbitacin E, and their glycosylated forms, including Arvenin I, Arvenin II, and Elaterinide. Additionally, the presence of Cucurbitacin S, although not yet characterized in these models, may further enhance this activity. The overall high relative abundance of Cucurbitacins, compounds with well-established cytotoxic properties, in both extracts likely explains why both P22 and P35 are active against all three cancer cell lines. These findings reinforce the therapeutic promise of Cucurbitacins as pharmacological agents in cancer prevention and treatment.

## 5. Conclusions

The combined morphological, physiological, and biochemical analyses of P35 *Ecballium elaterium* plants compared to their P22 counterparts reveal adaptive strategies that are both structural and metabolic in nature. Although no major and impressive anatomical changes were noted, P35 displayed significantly reduced dry mass and stomatal density, along with increased transition zone diameter—suggesting an internal compensation mechanism to counter heat-induced stress. In the leaves, MDA and H_2_O_2_ levels decreased under heat stress, while protective compounds such as proline (↑41.3%) and total phenolic content (↑23.9%) increased. This suggests that leaf tissues may have effectively mitigated oxidative stress through targeted protective responses. In contrast, the roots showed concurrent increases in MDA (↑28.4%), H_2_O_2_ (↑25.5%), and soluble sugars (↑24.1%), indicating a more integrated and resilient stress response. These tissue-specific dynamics were further illustrated by PCA, where the leaves showed compartmentalized responses, while the roots displayed co-regulated and broadly protective patterns, and a generally more stable stress response profile.

From a pharmacological standpoint, heat stress significantly influenced the phytochemical profile of *E. elaterium*, particularly increasing the glycosylated forms of bioactive Cucurbitacins in P35 leaves. These compositional shifts translated into distinct cytotoxic profiles against human cancer cell lines. To the best of our knowledge, this is the first time that leaf extracts from squirting cucumbers have been used in cell lines. Extracts from P35 demonstrated enhanced cytotoxicity against SH-SY5Y neuroblastoma cells and prostate (DU-145) cancer cells; meanwhile, P22 extracts exhibited anticancer activity, suggesting a neurotoxic effect, possibly driven by elevated glycosylated Cucurbitacins like Cucurbitacin B (Arvenin I) and Cucurbitacin E (Elaterinide). Conversely, MCF-7 breast cancer cells demonstrated a dose-dependent response to both P22 and P35 extracts. While P22 extracts exhibited slightly higher cytotoxicity at some concentrations (as reflected by a marginally lower IC_50_), P35 extracts induced comparable or greater cell death at other doses (e.g., 10 µg/mL and 1000 µg/mL). These results suggest that thermal stress may have modulated the bioactivity profile of the extracts, but not in a uniformly suppressive or enhancing manner also depending on the cancer type.

Analyses via LC-HRMS profiling confirmed Cucurbitacins and their glycosides as the principal bioactive constituents, with distinct compositions between P22 and P35. This remarkable biochemical plasticity underscores how environmental stress not only shapes plant morphology and physiology but also alters secondary metabolite profiles in ways that significantly impact bioactivity. Consequently, plants’ stress conditions may offer a novel approach both to plant stress biology and to optimizing the therapeutic potential of medicinal plants based on targeted cytotoxic applications.

## Figures and Tables

**Table 1:** The values for total dry mass as well as the thickness of the transition zone are presented. The measured values refer to the average from both experiments. (Asterisks indicate the statistical differences between treatments; *p* ≤ 0.05, according to Tukey’s test comparisons.)

Treatment (Both Experiments)	Total Dry Mass (g)	Transition Zone Diameter (mm)
*Ecballium elaterium*—P22	2.8 ± 0.4 *	6.96 ± 0.22 **
*Ecballium elaterium*—P35	1.7 ± 0.3 *	8.26 ± 0.56 **

**Table 2:** The number of stomata and glandular trichomes on adaxial and abaxial epidermis are displayed. The measured values refer to the average from both experiments. (Asterisks indicate the statistical difference between treatments; *p* ≤ 0.05, according to Tukey’s test comparisons.)

Treatment (Both Experiments)	Number of Stomata/mm^2^—Adaxial	Number of Stomata/mm^2^—Abaxial	Number of Glandular Trichomes/mm^2^ —Abaxial
*Ecballium elaterium*—P22	221 ± 28 *	N/A	29 ± 10 **
*Ecballium elaterium*—P35	119 ± 9 *	61 ± 19	2 ± 1.2 **

**Table 3:** UHPLC-HRMS/MS data of the *Ecballium elaterium* methanolic extracts’ annotated compounds.

ID	Annotated Compound	Rt (min)	Experimental *m*/*z* [M-H]^−^	Experimental *m*/*z* [M+H]^+^	Elemental Composition (EC)	RDB_eq._ Values	Δm (ppm)	HRMS/MS Fragments	References
1	Trehalose	0.92	377.0850 [M+Cl]^−^		C_12_H_22_O_11_	1.50	−1.73	341.1086 (100), 89.0243 (72), 179.0563 (72) 119.0352 (42), 161.0455 (33), 59.0140 (24), 101.0246 (24) 71.0139 (22), 113.0247 (20), 221.0670 (11)	-
2	Unknown	0.95		323.1447	C_12_H_22_N_2_O_8_	2.50	−0.64	239.1026 (100), 195.1127 (55), 287.1238 (47), 108.0443 (41), 150.0549 (40), 81.0335 (39), 115.0865 (37), 100.0756 (29), 225.1235 (24), 161.0920 (19), 173.0920 (15)	-
3	Unknown	1.23		323.1446	C_12_H_22_N_2_O_8_	2.50	−0.93	239.1026 (100), 195.1128 (55), 81.0334 (43), 287.1239 (40), 108.0443 (39), 150.0549 (36), 115.0865 (31), 100.0756 (30), 225.1233 (23), 161.0920 (16), 173.0921 (16), 122.0600 (15)	-
4	Citric acid	1.24	191.0195		C_6_H_8_O_7_	3.50	−0.94	111.0087 (100), 87.0088 (42), 85.0295 (28), 191.0198 (11)	[51]
5	Gentisic acid O-glycoside	1.91	315.0721		C_13_H_16_O_9_	6.50	−0.04	152.0115 (100), 315.0721 (74), 153.0194 (50), 108.0217, (31), 109.0295 (26)	-
6	Unknown	4.19	367.1605		C_15_H_28_O_10_	2.50	−1.22	235.1186 (100), 101.0244 (80), 59.0138 (46), 71.0138 (44), 89.0244 (42), 161.0455 (34),131.0350 (31), 113.0244 (26), 73.0295 (18), 85.0295 (16)	-
7	Megastigmane 9-O glucopyranoside	4.39	451.2180 [M+FA-H]^−^		C_19_H_34_O_9_	3.50	−1.17	167.1078 (100), 89.0246 (77), 71.0139 (41), 101.0245 (39), 59.0139 (36), 113.0247 (31), 149.0970 (25)	[52]
8	Kaempferol 3-O diglycoside	4.56	593.1515		C_27_H_30_O_15_	13.50	0.09	473.1093 (100), 503.1187 (36), 593.1496 (20)	[52]
9	Quercetin 3-O diglycoside	5.19	741.1876		C_32_H_38_O_20_	14.50	−1.09	300.0273 (100), 741.1880 (41), 301.0352 (13)	[52]
10	Luteolin 8-C glycoside	5.27	447.0933		C_21_H_20_O_11_	12.50	−0.53	327.0512 (100), 357.0611 (69), 285.0407 (10)	[53]
11	Rutin	5.64	609.1456		C_27_H_30_O_16_	13.50	−0.99	300.0273 (100), 609.1457 (63), 301.0352 (57)	[53]
12	Kaempferol 3-O diglycoside	5.99	593.1513		C_27_H_30_O_15_	13.50	0.19	285.0403 (100), 284.0327 (61), 593.1509 (45)	[53]
13	Narcissin	6.08	623.1616		C_28_H_32_O_16_	13.50	−0.12	315.0509 (100), 314.0431 (41), 623.1614 (20)	-
14	Khekadaengoside K	6.68	561.2701		C_30_H_42_O_10_	10.50	−0.68	561.2705 (100), 398.2093 (40), 399.2171 (35), 543.2598 (30)	[52]
15	Cucurbitacin J 2-O glucopyranoside	6.70	693.3488		C_36_H_54_O_13_	10.50	−0.59	605.2968 (100), 425.2329 (63), 675.3386 (28), 443.2439 (16), 101.0608 (14), 341.1765 (13), 383.2227 (13), 587.2856 (12), 513.2847 (12), 495.2741 (12), 657.3276 (11)	[54]
16	Cucurbitacin I isomer 1	6.70		515.3000	C_30_H_42_O_7_	9.50	−0.66	515.2998 (100), 91.0540 (18), 205.1222 (17), 111.0804 (16), 123.0802 (14), 95.0491 (14), 128.0618 (12), 115.0542 (12), 215.1428 (10), 139.0753 (10), 187.1117 (10)	[55]
17	Khekadaengoside D	7.15	721.3434 [M+FA-H]^−^		C_36_H_52_O_12_	11.50	−1.47	675.3384 (100), 657.3282 (26) 495.2751 (22), 513.2860 (14) 341.1757 (12) 477.2645 (12) 411.2178 (11)	[54]
18	Cucurbitacin S	7.17		499.3045	C_30_H_42_O_6_	9.50	−0.60	499.3045 (100), 113.0960 (62), 91.0541 (55), 123.0804 (44), 149.0960 (41), 95.0491 (40), 95.0854 (40), 128.0622 (31), 115.0542 (40), 67.0542 (27), 79.0542 (23), 111.0804 (22)	[56]
19	Arvenin III	7.41	723.3588 [M+FA-H]^−^		C_36_H_54_O_12_	10.50	−1.08	659.3441 (100), 575.2862 (99), 413.2334 (88) 87.0087 (87), 101.0244 (71), 89.0243 (64), 505.2449 (58), 113.0244 (55), 119.0350 (51), 497.2910 (51), 343.1914 (46), 641.3337 (45), 191.1081 (37), 677.3530 (35), 161.0453 (34), 165.0921 (31), 437.2694 (30), 455.2806 (29), 479.2805 (27), 485.2531 (25), 599.3241 (23), 617.3338 (22), 325.1815 (21), 547.2554 (20)	[57]
20	Unknown Ursane-type triterpenoid glycoside	7.60	707.3646 [M+FA-H]^−^		C_36_H_54_O_11_	10.50	−0.36	87.0089 (100), 101.0247 (95), 499.3076 (84), 661.3618 (77), 571.3281 (77), 191.1081 (71), 161.0458 (68), 113.0247 (47), 309.2073 (33), 89.0244 (33), 448.8047 (30), 119.0352 (29), 135.6041 (27)	-
21	Unknown Lanostane-type triterpenoid	7.81	547.2910		C_30_H_44_O_9_	9.50	−0.55	135.0815 (100), 331.1915 (18), 153.0921 (9), 485.2908 (9)	-
22	Arvenin I Isomer 1	7.97	765.3698 [M+FA-H]^−^		C_38_H_56_O_13_	11.50	−0.63	701.3542 (100), 497.2903 (31), 719.3652 (30), 101.0244 (27), 659.3442 (24), 87.0087 (22), 573.2700 (21), 161.0455 (16), 113.0244 (16), 89.0244 (15)	[54]
23	Elaterinide	8.31	763.3542 [M+FA-H]^−^		C_38_H_54_O_13_	-1.59	12.50	657.3273 (100), 495.2751 (43), 717.3499 (34), 615.3170 (23), 561.2706 (17), 675.3434 (14), 477.2666 (11), 699.3367 (11)	[52]
24	Arvenin I Isomer 2	8.45	765.3696 [M+FA-H]^−^		C_39_H_58_O_15_	11.50	−0.87	701.3543 (100), 497.2909 (32), 719.3641 (27), 659.3430 (26), 101.0244 (24), 87.0088 (22), 113.0245 (17)	
25	Arvenin II	8.58	767.3851 [M+FA-H]^−^		C_38_H_58_O_13_	10.50	−1.06	643.3481 (100), 481.2959 (85), 609.2919 (82), 661.3586 (80), 703.3665 (67), 101.0244 (48), 87.0087 (39), 161.0455 (37), 553.3176 (33), 113.0243 (29), 89.0246 (22), 541.3177 (20),	[55]
26	Cucurbitacin R	8.64	561.3063 [M+FA-H]^−^		C_30_H_46_O_7_	8.50	−0.59	165.0921 (100), 385.2382 (93), 369.2068 (77), 59.0138 (66), 179.1078 (48), 499.3072 (44), 137.0973 (42), 457.2961 (42), 367.2273 (41), 325.1810 (41), 341.2112 (29), 173.1184 (16), 439.2820 (16)	[54]
27	Deoxocucurbitoside B	8.75	835.4116 [M+FA-H]^−^		C_42_H_62_O_14_	12.50	−0.72	789.4066 (100), 481.2956 (32), 505.2957 (22), 480.2877 (19), 101.0242 (12)	[52]
28	Dihydro-Cucurbitacin D isomer 1	8.78	561.3063 [M+FA-H]^−^		C_30_H_44_O_7_	9.50	0.88	165.0921 (100), 497.2917 (44), 439.2494 (40), 479.2809 (36), 437.2708 (31), 515.3033 (30), 385.2021 (25), 455.2806 (24), 385.2386 (22), 369.2079 (20), 497.2515 (18), 343.1927 (17), 87.0454 (10)	[54]
29	Dihydro-Cucurbitacin D isomer 2	8.94	561.3063 [M+FA-H]^−^		C_30_H_44_O_7_	9.50	−0.54	165.0920 (100), 479.28109 (36), 437.2702 (32), 497.2921 (29), 455.2823 (28), 439.2477 (22), 385.2008 (18)	[56]
30	Seco-Cucurbitacin B derivative	9.05	589.3016		C_32_H_46_O_10_	10.50	−0.42	135.0815 (100), 331.1913 (17), 485.2906 (8), 467.2805 (6)	[54]
31	Cucurbitacin I isomer 3	9.16	559.2906 [M+FA-H]^−^		C_30_H_44_O_7_	9.50	−0.66	163.0766 (100), 497.2905 (71), 383.2230 (62), 59.0138 (42), 203.1081 (33), 367.1915 (32), 179.1081 (30), 455.2811 (27), 437.2665 (22), 339.1970 (21), 173.1189 (21)	-
32	Unknown (Sphingolipid)	9.38		288.2896	C_17_H_37_NO_2_	−0.50	−0.96	288.2898 (100), 106.0862 (40), 88.0757 (21), 57.0699 (14)	-
33	Unknown (Sphingolipid)	9.58		288.2891	C_17_H_37_NO_2_	−0.50	−2.13	288.2893 (100), 88.0756 (12), 106.0861 (9)	-
34	Unknown (Sphingolipid)	9.71		304.2844	C_17_H_37_NO_3_	−0.50	−0.85	304.2843 (100), 256.2633 (57), 88.0756 (21) 56.0494 (14), 74.0600 (12), 122.0810 (10)	-
35	Cucurbitacin B	10.18	603.3168 [M+FA-H]^−^		C_32_H_46_O_8_	10.50	−1.06	497.2909 (100), 411.2176 (90), 301.1445 (34), 273.1486 (19)	[52]
36	Unknown (Sphingolipid)	10.42		316.3206	C_19_H_41_NO_2_	−0.50	−1.23	316.3206 (100), 106.0861 (31), 88.0756 (19), 57.0699 (12)	-
37	Cucurbitacin E	10.76	601.3015 [M+FA-H]^−^		C_32_H_44_O_8_	11.50	−0.41	409.2027 (100), 495.2733 (96), 163.0764 (93), 299.1280 (53), 463.7701 (51), 426.2051 (47), 299.0011 (41), 165.9267 (41), 175.3833 (40), 195.3096 (39), 87.3690 (39), 322.1431 (39)	[52]
38	Gingerglycolipid A-Glycerolipid	11.07	721.3643 [M+FA-H]^−^		C_33_H_56_O_14_	6.50	−1.52	277.2171 (100), 397.1364 (37), 101.0247 (24), 415.1458 (19), 89.0245 (18)	-
39	LysoPC (18:3)-Glycerophospholipid	11.24	562.3146 [M+FA-H]^−^		C_26_H_48_NO_7_P	4.50	−0.83	277.2173 (100), 502.2940 (12), 224.0692 (11)	-
40	MGMG (18:3)-Glycolipid	12.15	559.3120 [M+FA-H]^−^		C_27_H_46_O_9_	5.50	−0.64	277.2172 (100), 253.0928 (11), 101.0245 (3), 278.2206 (3)	-
41	DGMG(16:0)-Glycolipid	12.30	653.3747 [M+FA-H]^−^		C_31_H_58_O_14_	3.50	−1.05	255.2328 (100), 89.0244 (31), 101.0244 (30), 397.1349 (23), 71.0138 (16), 415.1457 (15)	-
42	LysoPC (16:0)-Glycerophospholipid	12.51	540.3303		C_25_H_52_NO_9_P	1.50	0.80	255.2328 (100), 480.4096 (17), 224.0693 (10)	-
43	PC(16:0/9:0(COOH))-Glycerophospholipid	13.80		666.4337	C_33_H_64_NO_10_P	2.50	−0.52	184.0734 (100), 86.0964 (21), 98.9842 (16), 124.9998 (10), 71.0730 (5)	-
44	SQMG(18:3)-Glycerolipid	14.60	577.2684		C_27_H_46_O_11_S	5.50	−0.75	577.2684 (100), 225.0074 (13), 64.1417 (5)	-
45	Unknown (Triterpenoid)	14.73		485.2894	C_29_H_40_O_6_	9.50	−0.82	133.1012 (100), 91.0542 (36)	-
46	Unknown (Sphingolipid)	15.31		526.5188	C_33_H_67_NO_3_	0.50	−0.62	270.2790 (100), 283.2630 (97), 526.5189 (94), 88.0756 (18), 102.0913 (12)	-
47	Monopalmitin	15.87		331.2841	C_19_H_38_O_4_	0.50	−0.51	57.0699 (100), 71.0855 (70), 95.0855 (54), 85.1012 (40), 109.1012 (26), 81.0698 (26), 313.2741 (22), 83.0855 (20), 67.0542 (18), 69.0699 (16), 55.0542 (12), 97.1011 (11)	-
48	Pheophorbide a	16.07		593.2751	C_35_H_36_N_4_O_5_	19.50	−1.18	593.2753 (100), 533.2546 (23), 460.2255 (9), 461.2329 (6), 505.2217 (6)	-
49	Monostearin	17.19		359.3155	C_21_H_42_O_4_	0.50	−0.18	57.0698 (100), 116.0527 (81), 71.0854 (70), 59.9902 (69), 95.0855 (52), 85.1012 (38), 88.0215 (37), 67.0543 (29), 81.0698 (28), 341.3047 (25), 109.1014 (25), 55.0541 (24), 69.0699 (23), 83.0855 (22), 57.0335 (13), 97.1009 (12), 158.1506 (11), 229.3898 (11), 111.1167 (10)	-

Rt: retention time expressed in minutes; RDB_eq_: ring and double bond equivalent; Δm: mass accuracy expressed in ppm.

## Data Availability

The original contributions presented in this study are included in the article/Supplementary Materials. Further inquiries can be directed to the corresponding author.

## Supplementary Materials

The following supporting information can be downloaded at: https://www.mdpi.com/article/10.3390/metabo15090585/s1, Figure S1: Ombrothermic diagram of the year 2022 for the Island of Ios, Cyclades, Greece (above). Temperature data for July and August from 2018 to 2023, the sampling year (below).
