# Supplementary material for: Ecballium elaterium (L.) A. Rich. (Squirting Cucumber) Plants Cultured Under Different Temperatures: Anatomical and Biochemical Modifications of Their Leaves and the Bioactivity of Leaf Extracts
Source: Metabolites. 2025 Aug 31;15(9):585. doi: 10.3390/metabo15090585 (PMC12471846; doi:10.3390/metabo15090585)
Supplement: Supplementary file 1 [file metabolites-15-00585-s001.zip › metabolites-3796581 Supplementary Material.pdf]

metabolites

MDPI

Article

Ecballium elaterium (L.) A. Rich. (Squirting Cucumber) Plants Cultured Under Different Temperatures: Anatomical and Biochemical Modifications of Their Leaves and the Bioactivity of Leaf Extracts

Aikaterina L. Stefi [1], Maria Chalkiadaki [2], Emily Bashari [1], Konstantina Mitsigiorgi [1], Paweł Szczeblewski [3], Danae Papageorgiou [1], Dimitrios Gkikas [1], Dido Vassilacopoulou [4], Nikolaos S. Christodoulakis [1] and Maria Halabalaki [2,*]

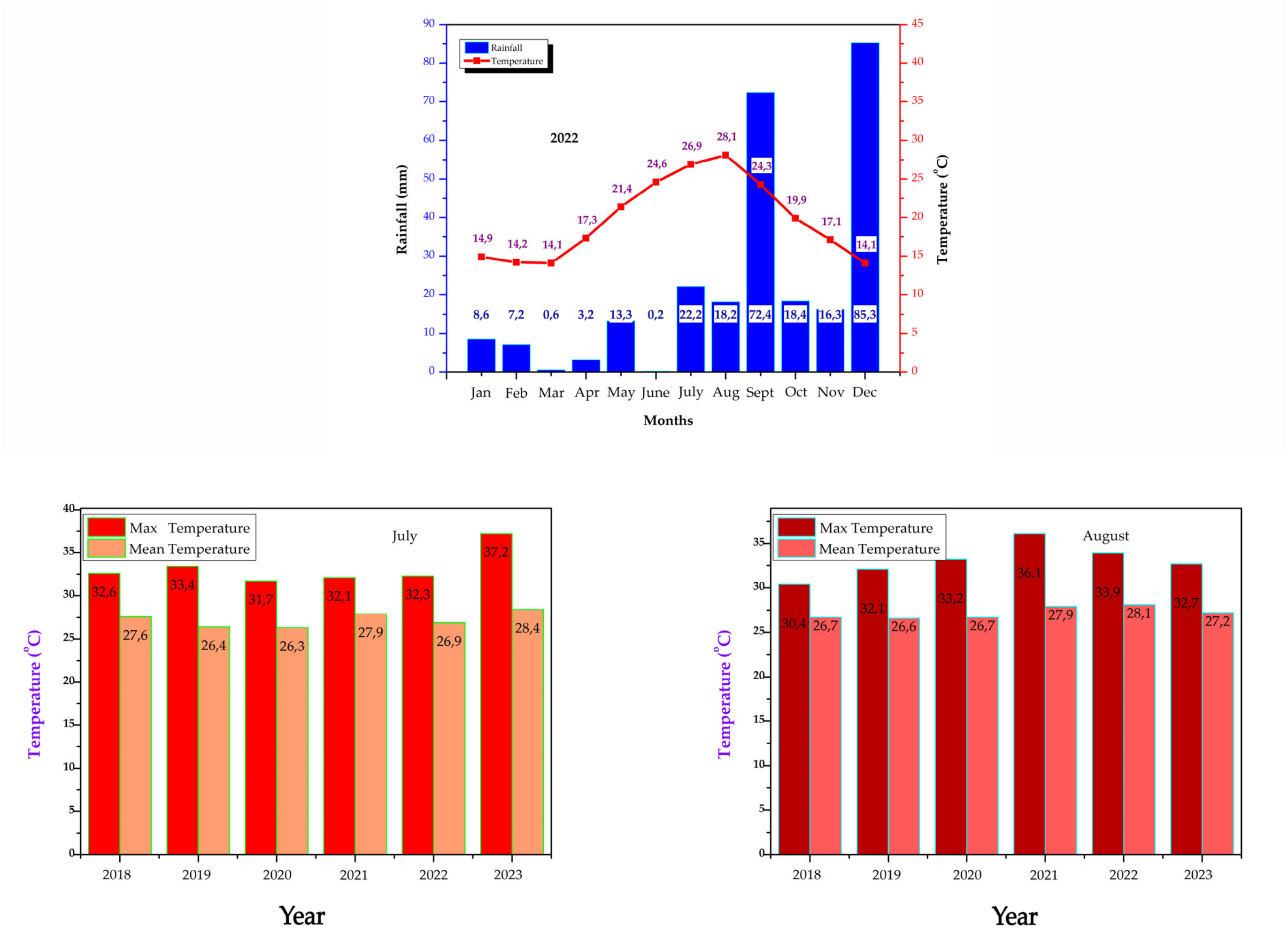

Supplementary Figure S1. Ombrothermic diagram of the year 2022 for the Island of Ios, Cyclades, Greece (above). Temperature data for July and August from 2018 to 2023, the sampling year (below).
